# Population genetic structure, antibiotic resistance, capsule switching and evolution of invasive pneumococci before conjugate vaccination in Malawi

**DOI:** 10.1016/j.vaccine.2017.07.009

**Published:** 2017-08-16

**Authors:** Chrispin Chaguza, Jennifer E. Cornick, Cheryl P. Andam, Rebecca A. Gladstone, Maaike Alaerts, Patrick Musicha, Chikondi Peno, Naor Bar-Zeev, Arox W. Kamng'ona, Anmol M. Kiran, Chisomo L. Msefula, Lesley McGee, Robert F. Breiman, Aras Kadioglu, Neil French, Robert S. Heyderman, William P. Hanage, Stephen D. Bentley, Dean B. Everett

**Affiliations:** a Department of Clinical Infection, Microbiology and Immunology, Institute of Infection and Global Health, University of Liverpool, Liverpool, UK; b Malawi-Liverpool-Wellcome Trust Clinical Research Programme, Blantyre, Malawi; c Center for Communicable Disease Dynamics, Department of Epidemiology, Harvard T.H. Chan School of Public Health, Boston, MA, USA; d Pathogen Genomics, Wellcome Trust Sanger Institute, Hinxton, Cambridge, UK; e Department of Biomedical Sciences, University of Malawi College of Medicine, Blantyre, Malawi; f Respiratory Diseases Branch, Centers for Disease Control and Prevention, Atlanta, USA; g Hubert Department of Global Health, Rollins School of Public Health, Emory University, Atlanta, USA; h Division of Infection and Immunity, University College London, London, UK

**Keywords:** *Streptococcus pneumoniae*, Antibiotic resistance, Population structure, Evolution, Capsule switching

## Abstract

**Introduction:**

Pneumococcal infections cause a high death toll in Sub Saharan Africa (SSA) but the recently rolled out pneumococcal conjugate vaccines (PCV) will reduce the disease burden. To better understand the population impact of these vaccines, comprehensive analysis of large collections of pneumococcal isolates sampled prior to vaccination is required. Here we present a population genomic study of the invasive pneumococcal isolates sampled before the implementation of PCV13 in Malawi.

**Materials and methods:**

We retrospectively sampled and whole genome sequenced 585 invasive isolates from 2004 to 2010. We determine the pneumococcal population genetic structure and assessed serotype prevalence, antibiotic resistance rates, and the occurrence of serotype switching.

**Results:**

Population structure analysis revealed 22 genetically distinct sequence clusters (SCs), which consisted of closely related isolates. Serotype 1 (ST217), a vaccine-associated serotype in clade SC2, showed highest prevalence (19.3%), and was associated with the highest MDR rate (81.9%) followed by serotype 12F, a non-vaccine serotype in clade SC10 with an MDR rate of 57.9%. Prevalence of serotypes was stable prior to vaccination although there was an increase in the PMEN19 clone, serotype 5 ST289, in clade SC1 in 2010 suggesting a potential undetected local outbreak. Coalescent analysis revealed recent emergence of the SCs and there was evidence of natural capsule switching in the absence of vaccine induced selection pressure. Furthermore, majority of the highly prevalent capsule-switched isolates were associated with acquisition of vaccine-targeted capsules.

**Conclusions:**

This study provides descriptions of capsule-switched serotypes and serotypes with potential to cause serotype replacement post-vaccination such as 12F. Continued surveillance is critical to monitor these serotypes and antibiotic resistance in order to design better infection prevention and control measures such as inclusion of emerging replacement serotypes in future conjugate vaccines.

## Highlights

• High pneumococcal population diversity in terms of serotypes and sequence types (ST).
• Decline in IPD incidence pre-vaccination not associated with specific serotypes.
• High prevalence and antibiotic resistance rates in serotype 1 isolates.
• High levels of capsule (serotype) switching pre-vaccination.
• Surveillance remains crucial to understand pneumococcal epidemiology.

## 1 Introduction

With over one million deaths and approximately fifteen million disease episodes annually, *Streptococcus pneumoniae* (the pneumococcus), is one of the most significant global causes of serious human infections including pneumonia, bacteremia and meningitis [1]. The highest burden and mortality due to invasive pneumococcal disease (IPD) occurs in resource poor settings such as Sub-Saharan Africa (SSA). In Malawi, it is the highest cause of bacterial meningitis [2], [3] and the second highest cause of bacteremia [4]. The incidence of adult IPD is estimated at 58 per 100,000 with the highest rates (108 per 100,000) recorded in adults aged between 35 and 40 due to high HIV prevalence [5] and rates in children substantially higher than this based on hospitalization data [6]. Nasopharyngeal carriage rates have been reported as 20% in adults [7] and 42% in children [8] often involving simultaneous carriage with multiple serotypes [9].

The heptavalent pneumococcal conjugate vaccine (PCV7), licensed in 2000 (www.gavi.org) targeted the seven most prevalent serotypes in the US out of nearly 100 serotypes characterized globally [10] and is highly effective against vaccine type IPD [11]. In contrast with the US, there was low theoretical serotype coverage in SSA (e.g. 40% in Malawi) due to dominance of non-PCV7 targeted serotypes particularly serotype 1 [8], [12]. Despite the high efficacy of PCV7 [13], following vaccination non-vaccine serotypes became more common in carriage and IPD, a phenomenon termed serotype replacement [14]. To guard against the emerging replacement serotypes such as 19A [14] and expand serotype coverage, higher valency PCVs (PCV10 and PCV13) were licensed. PCV13 was introduced in Malawi in 2011 [15], which targets serotypes 1, 3, 4, 5, 6A, 6B, 7F, 9V, 14, 18C, 19A, 19F and 23F.

Given that changes in the pneumococcal genome particularly the capsule biosynthesis genes could impact effectiveness of pneumococcal conjugate vaccine formulations, a crucial component of ensuring sustained prevention of pneumococcal disease will be monitoring pneumococcal genomic and phenotypic evolution over time. In Malawi, previous work investigated the genetic structure of the invasive isolates [16] but due to limitations including a smaller dataset (n = 134), it was not adequate to effectively resolve the genetic structure and temporal evolution of the pneumococcal lineages. Here we extend this analysis to conduct a population genomic analysis of whole genome sequenced invasive isolates (n = 585) sampled over a seven-year period (2004–2010) before the implementation of PCV13 vaccine in November 2011 in Malawi. Due to the fact that pneumococci frequently switch their serotype by swapping genes between different serotypes involved in capsule biosynthesis, we analysed the serotype and lineage distribution, antibiotic resistance, temporal evolution and capsule switching in context of genetic structure of the isolates.

## 2 Materials and methods

We retrospectively sampled 585 invasive pneumococcal isolates from blood and cerebral spinal fluid (CSF) from the bacterial isolate archive at the Malawi-Liverpool-Wellcome Trust Clinical Research Programme for whole genome sequencing (Supplemental Table S1). The isolates were sampled blindly of the serotype in order to represent their prevalence in IPD and not based on inclusion based on their serotypes. The isolates in the archive were collected from patients at the Queen Elizabeth Central Hospital in Blantyre, the largest referral hospital in Southern Malawi. We extracted DNA using QIAamp DNA mini kit, QIAgen Biorobot (Qiagen, Hilden, Germany), and Wizard® DNA Genomic DNA Purification Kit (Promega, WI, USA). DNA sequencing was done at the Wellcome Trust Sanger Institute using Illumina Genome Analyzer II and HiSeq platforms (Illumina, CA, USA). Whole genome alignment, sequence assembly, phylogeny construction, recombination detection, detection of antibiotic resistance genes, coalescent, and statistical analyses were done as described in Supplemental Materials and Methods. Sequence typing and serotyping were done using multilocus sequence typing (MLST) [17], [18], and PCR [19] and genomic approach respectively [20]. The sequence reads for the isolates were deposited in the European Nucleotide Archive (www.ebi.ac.uk/ena) and their accession numbers are provided in Supplemental Table S1. We used disc diffusion for antibiotic susceptibility testing and interpreted the findings using the British Society Antimicrobial Chemotherapy (BSAC) guidelines. The study was approved by the University of Malawi’s College of Medicine Research and Ethics Committee (approval number: P08/14/1614).

## 3 Results

### 3.1 Characteristics of pneumococcal isolates before vaccination

**Fig. 1:**
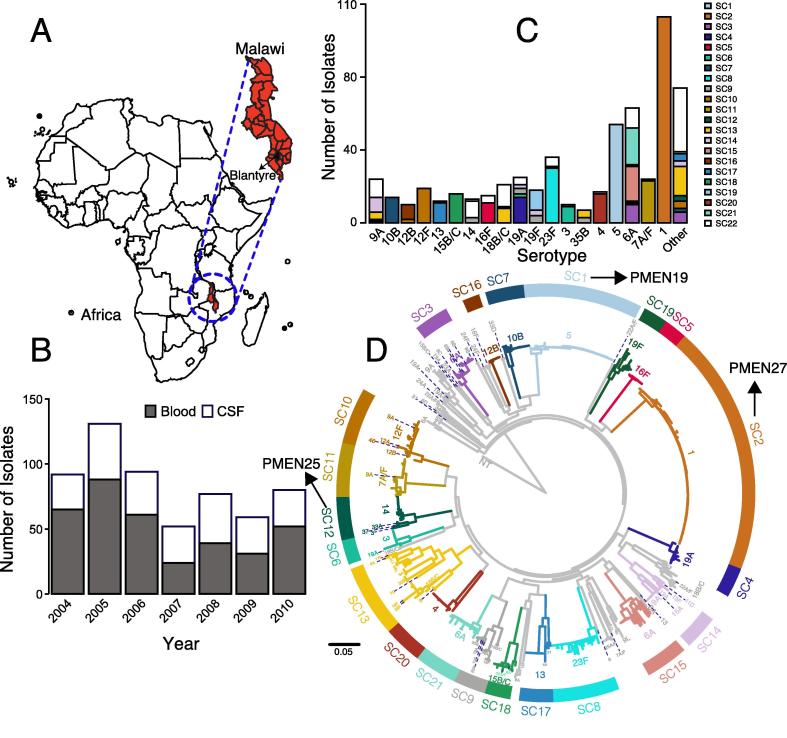
Pneumococcal genetic population structure pre-vaccination in Malawi. (A) Map of Africa and Malawi showing the sampling location of the isolates. (B) Number of pneumococcal isolates from blood and cerebrospinal fluid (CSF) sequenced every year. (C) Stacked bar plot showing number of isolates of in serotypes with at least 3% prevalence. All other serotypes were grouped as ‘other’. The bars are categorized by sequence cluster (SC). (D) Maximum likelihood phylogeny annotated with the sequence clusters (SCs) showing genetic relationships of the isolates. Both SCs and serotypes are labelled on the tree and branches for the monophyletic SCs (SC1-21) are colored in non- colors while SC22 (polyphyletic clade) is colored in . The phylogeny was out-group rooted using a classical non-typeable (NT) isolate obtained from carriage.

Pneumococcal isolates from blood and CSF were collected from adults and children through routine pathogen surveillance at the Queen Elizabeth Central Hospital, the largest referral hospital in Blantyre, Malawi. We sequenced a randomly sample of 585 isolates from collection of >5000 pneumococcal isolates from 2004 to 2010 for whole genome sequencing in order to determine the pneumococcal genomic epidemiology and evolution pre-PCV13 implementation in 2011 (Fig. 1A and B and Table S1). Of these samples, 65.5% and 38.5% of the isolates were from blood and cerebrospinal fluid (CSF) respectively. By vaccine status, 68.7% of the study isolates contained a vaccine type (VT) serotype targeted by the PCV13 vaccine formulation. Although the number of isolates collected were higher in children <5 years old and adults above 30 years old, the prevalence of vaccine type (VT) serotypes decreased consistently with increasing age (Fig. S1A–C). The prevalence of serotypes also varied by these age groups, with some serotypes common in the under fives.

### 3.2 Genetic population structure analysis reveals high population diversity

To determine the pneumococcal population structure and diversity, we did comparative genomic analysis of the isolates by clustering the isolates into sequence clusters (SC) using an unsupervised Bayesian hierarchical clustering approach [21]. Such SCs defines the unique subpopulations of genetically similar isolates, which are predominantly of the same serotype but some SCs contained multiple serotypes because of serotype switching due to recombination-mediated swapping of genes between isolates of different capsule types (Fig. 1C). Overall 22SCs were identified and of these 22 SCs, SCs 1–21 were monophyletic with a single common ancestor while SC22 had multiple common ancestors and thus it was polyphyletic (Fig. 1D). Due to the inclusion of more isolates in this study, the number of SCs identified were identified than in a previous study [22]. Because of the high sequence diversity in SC22, our analysis of SCs focuses largely on SCs 1–21. Overall, the sequenced samples were comprised of 46 serotypes and 134 sequence types (ST).

### 3.3 Prevalence of pneumococcal serotypes and SCs – high dominance of serotype 1 lineage

**Fig. 2:**
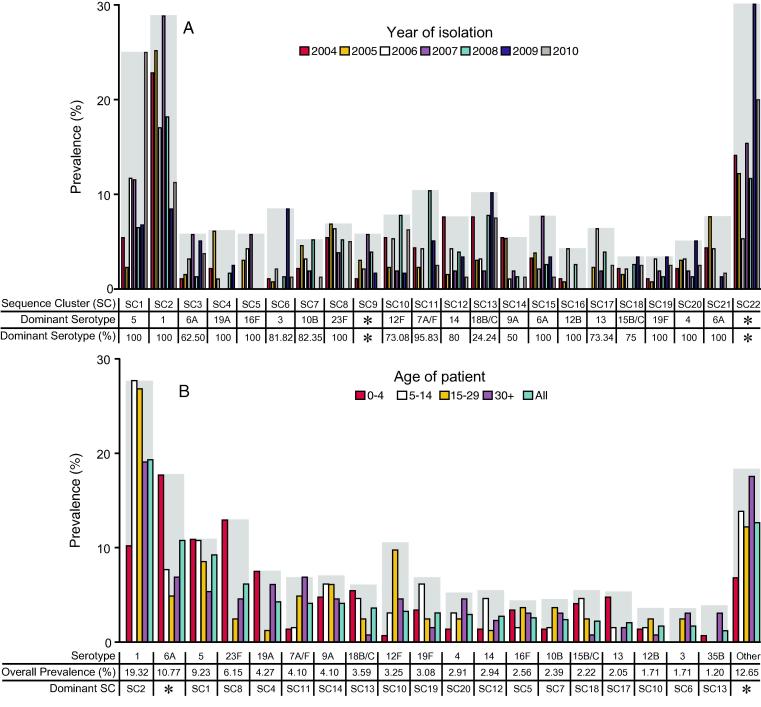
Prevalence of pneumococcal sequence clusters (SCs) and serotypes. (A) Annual prevalence of SCs and prevalence of dominant serotype in the SCs. (B) Prevalence of pneumococcal serotypes by age groups.

The most dominant monophyletic SC was SC2 and was comprised of only serotype 1 isolates (19.3%) which are mostly (84.96%) of multi locus sequences type 217 (ST217, also known as Sweden^1^-27 or PMEN27) [23], [24] (Figs. 1C, 2A). The prevalence of serotype 1 peaked in adolescents (Fig. 2B). Other dominant serotypes included serotype 6A (10.8%) in multiple SCs including SC3 (ST2902), SC15 (ST2285/ST9532) and SC21 (ST2987), serotype 5 (9.2%) in SC1 (ST289) and serotype 23F (6.2%) in SC8 (ST802). Other SCs with only a single serotype included serotypes 5 in SC1 (ST289) or Colombia^5^-19 (PMEN19) [25], 16F in SC5 (predominantly ST705), 19A in SC4 (ST9457), 10B in SC7 (ST7055), 23F in SC8 (ST802), 6A in SC15 (ST9532), 12B in SC16 (ST10583), 4 in SC20 (ST2213) and 6A in SC21 (ST2987). Other SCs contained multiple serotypes such as SC12, a PMEN25 (Sweden^15A^-25) lineage, which contained ST63 isolates revealing a serotype switch in Malawi from serotype 15A to serotype 14 [26]. The annual prevalence of serotypes was stable temporally although prevalence of serotype 1 appeared to decline but this was likely due to sampling bias. Conversely, prevalence of serotype 5, which was largely from 2004 to 2009 showed an increased prevalence in 2010, which suggested the occurrence of an undetected small-scale local outbreak in 2010 (Fig. S2).

### 3.4 Antibiotic resistance rates in different serotypes

**Fig. 3:**
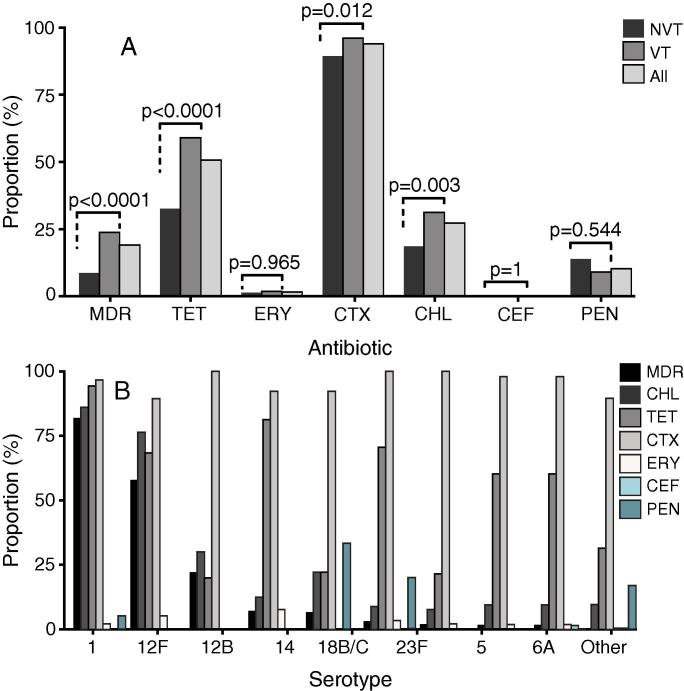
Phenotypic antibiotic resistance in invasive pneumococcal isolates in Malawi. (A) Antibiotic resistance in vaccine type (VT) and non-vaccine types (NVT). (B) Resistance rates for different pneumococcal serotypes. Resistance rates are shown for tetracycline (TET), chloramphenicol (CHL), cotrimoxazole (CTX), erythromycin (ERY), ceftriaxone (CEF), penicillin (PEN) and multidrug resistance (MDR).

Phenotypically, antibiotic resistance rates were variable with highest rates associated with cotrimoxazole (93.91%) followed by tetracycline (50.63%), chloramphenicol (27.29%) and penicillin (10.37%) while low resistance was associated with erythromycin (1.6%) and ceftriaxone (0.38%) (Fig. 3A). Because of limited or unavailability of phenotypic data, resistance rates for cefaclor and ampicillin are not presented. Highest multidrug resistance (MDR) rate was observed among the serotype 1 isolates (81.91%), which harbored isolates mostly resistant to tetracycline (94.34%), cotrimoxazole (96.74%) and chloramphenicol (86.11%). Overall, there was higher resistance among VT than NVT isolates for tetracycline (*p* *<* 0.0001), chloramphenicol (*p* = 0.003) and cotrimoxazole (*p* = 0.01) but not the other antibiotics although prevalence of penicillin resistant isolates appeared to be slightly higher in NVTs than for VTs (Fig. 3A). Serotype 12F (SC10), an NVT serotype, showed the second highest MDR rate (57.9%) after serotype 1 isolates (Fig. 3B).

### 3.5 Most pneumococcal lineages showed recent emergence

**Fig. 4:**
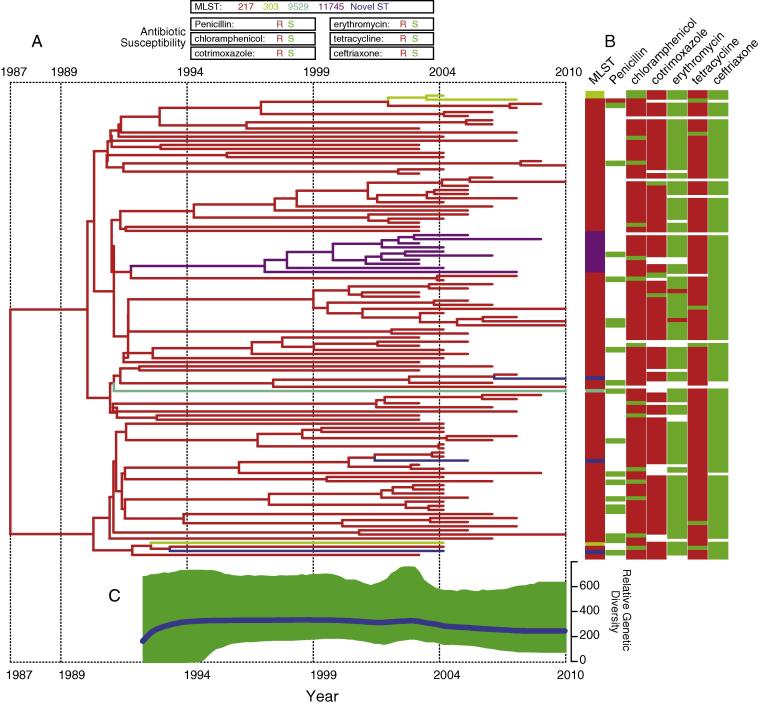
Temporal evolution of serotype 1 (SC2) isolates in Malawi. (A) Time-dated phylogeny of the serotype 1 isolates in clade SC2 showing the times of emergence of the clades and genetic similarities of the isolates. (B) Multilocus sequence typing (MLST) and antibiotic susceptibility profiles of the isolates in the phylogeny. Absence of antibiotic susceptibility data is presented by a color in the columns at the tip of the phylogeny. The key on top of the phylogeny shows the MLST profiles and antibiotic susceptibility – resistance (R) and susceptible (S). (C) Bayesian Skyline plot (BSP) showing changes in the relative genetic diversity, which corresponds to the effective population size of the isolates Prevalence of isolates. The blue line shows the mean while the green band represents the 95% credible interval. (For interpretation of the references to color in this figure legend, the reader is referred to the web version of this article.)

Where we were able to calibrate a molecular clock, we estimated the time of emergence and mutation rates for the SCs using BEAST [27]. Only five SCs namely SC1, SC2, SC3, SC5 and SC11 revealed sufficient molecular-clock signal and were used for coalescent analysis (Fig. S3). The mean mutation rates for the SCs ranged from 6.46 × 10^−06^ to 1.13 × 10^−05^ SNPs/site/year, which equated to the introduction of one to as high as twenty-five SNPs in the genomes per year (Fig. S4). Serotype 1 in SC2 was highly clonal and coalescent analysis showed that it emerged recently ∼1987 (95% credible interval [CI]:1981–1992) and was the ancestral serotype 1 ST in Malawi (Fig. 4). Since the emergence of SC2, serotype 1 isolates have shown high stability in their relative genetic diversity (or the effective population size) with no observable changes in antimicrobial resistance rates. Clade SC5, which contains serotype 16F showed the most recent emergence (∼2004) while the other SCs emerged in 1980s similarly to SC2 (Fig. S3). Serotype 5 isolates in SC1 dates back to ∼1983 (95% CI:1971–1992), SC3 (6A) in ∼1988 (95% CI:2002–1961), serogroup 7 (7A/F) isolates in SC11 emerged in ∼1970 although this may not be very reliable due to the large confidence intervals (95% CI:1845–1998).

### 3.6 High occurrence of non-PCV13 induced capsule switching

**Fig. 5:**
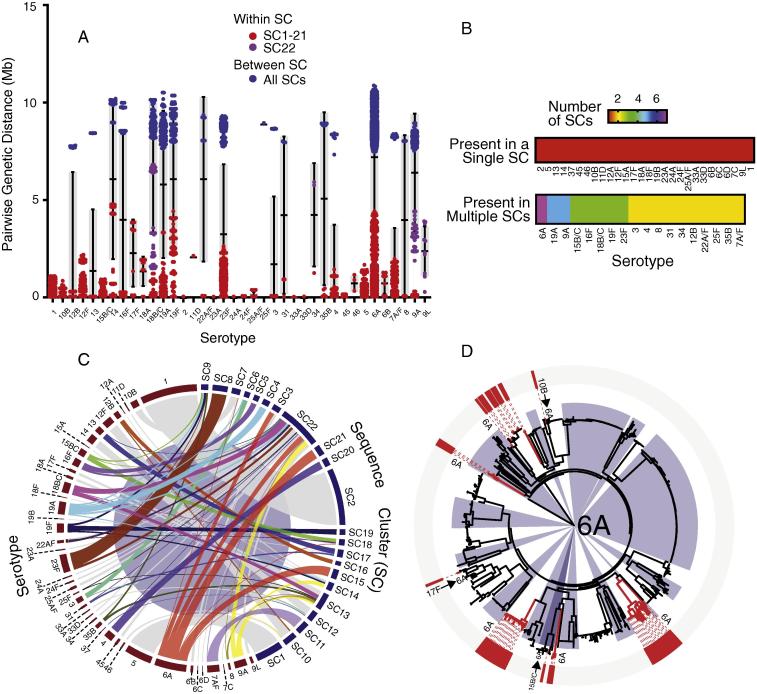
Within serotype sequence variation and association of capsules (serotypes) with lineages. (A) Pairwise differences measured as number of single nucleotide polymorphisms (SNPs) between isolate pairs of the same serotype are shown in red. Differences between isolates in SC22 are shown in pink while blue represents differences between isolates from different SCs (including SC22). (B) Number of SCs associated with each serotype. (C) Distribution of serotypes across the SCs showing serotypes uniquely associated with either single or multiple SCs (serotype switching). (D) Distribution of the serotype 6A capsule showing its multiple acquisitions across the phylogeny. (For interpretation of the references to color in this figure legend, the reader is referred to the web version of this article.)

Pneumococcal isolates can switch their serotype (capsule) through mutations and recombination in the capsule biosynthesis locus [28]. Certain serotypes particularly those with high intra-serotype sequence diversity such as serotypes 6A, 19A, 18B/C and those associated with more SCs were associated with multiple SCs due to non-PCV13 induced capsule switching (Fig.5A–C). Capsule-switched serotypes were inferred as the isolates with identical STs but different serotypes and in some case genetically related isolates with different STs but identical serotypes. Because a serotype is a derived trait (phenotype), assigning directionality of a serotype switch was based on either the genetic relatedness of the switched strains or their prevalence whereby the acquired capsule was already associated with another dominant lineage. Occurrence of capsule switching between very closely related isolates reflected their recent occurrence as such the potential original serotypes could be inferred (Table 1). On the other hand, the original serotypes could not be determined for non-recent switches especially where there was replacement of the original serotype in the SCs due to successful clonal expansion of the capsule-switched serotype. Examples of non-recent capsule switches included acquisitions of serotype 6A in multiple SCs such as SC3, SC15 and SC21 (Fig. 5D, Fig. S5). Majority of the recent capsule switches occurred not between isolates of the same ST (e.g. ST361^17F^ → ST361^6A^ in SC13) but also of the same serogroup (e.g. ST989^12F^ → ST989^12B^ in SC10) (Table 1). Serotype within identical serogroups reflected occurrences of spontaneous mutations in the capsule biosynthesis locus (e.g. ST2902^6A^ → ST2902^6B^ in SC3) while recombination caused switches between serogroups (e.g. ST5080^23A^ → ST5080^9A^ in SC22). Although occurrence of capsule-switches did not vary between VT and NVT serotypes, switches to VT serotypes appeared to result in higher prevalence of the capsule-switched isolates than NVT serotypes (Table 1).

**Table 1:** Recent capsule switches in pneumococcal isolates pre-vaccination in Malawi.

Serotype switch	Sequence cluster (SC)	Sequence type		Same STs	Same serogroup	Vaccine status	
		Previous serotype	Switched serotype			Previous serotype	Switched serotype
6A → 6C	SC3	ST9466	ST9466	Y	Y	VT	NVT
6A → 6B	SC3	ST2902	ST2902	Y	Y	VT	VT
35B → 23F	SC9	ST361	ST361	Y	N	NVT	VT
35B → 19F	SC9	ST361	ST361	Y	N	NVT	VT
35B → 19A	SC9	ST361	ST10599	Y	N	NVT	VT
7A/F → 9A	SC11	ST8672	ST9531	N	N	VT	NVT
12F → 12B	SC10	ST989	ST989	Y	Y	NVT	NVT
12F → 46	SC10	ST989	ST9544	N	N	NVT	NVT
12F → 12A	SC10	ST989	ST989	N	Y	NVT	NVT
12F → 9A	SC10	ST989	ST989	Y	N	NVT	NVT
18B/C → s18F	SC13	ST9523	ST9523	Y	Y	VT	NVT
18B/C → 25F	SC13	ST9523	ST9523	Y	N	VT	NVT
17F → 6A	SC13	ST9926	ST9926	Y	N	NVT	VT
15B/C → 6A	SC18	ST9572	ST9572	Y	N	NVT	VT
15B/C → 22A/F	SC18	ST9936	ST9936	Y	N	NVT	NVT
18B/C → 22A/F	SC22	ST5266	ST5266	Y	N	VT	NVT
18B/C → 9A	SC22	ST5266	ST5266	N	N	VT	NVT
23A → 9A	SC22	ST5080	ST5080	Y	N	NVT	NVT

## 4 Discussion

In this study we demonstrate the pneumococcal genetic population structure before introduction of PCV13 vaccine in Malawi. We found a high serotype and clonal diversity in Malawi and dominance of serotype 1 lineage (SC2), which was associated with highest antibiotic resistance rates. The prevalence of other serotypes remained stable although there was an increase in prevalence of PMEN19 isolates (SC1) in 2010. We also showed recent important or emergence of different pneumococcal lineages and serotypes using coalescent analysis, and high levels of recombination-mediated natural capsule switching in the absence of vaccine induced selection pressure. Some capsule switched lineages underwent successful clonal expansion over time resulting in the formation of multiple lineages with identical serotypes.

Serotype 1 (SC2) is common in SSA hence its dominance in Malawi to cause 19.3% of the IPD was not unexpected [29], [30], [31], [32]. Other serotypes such as serotype 5 (SC1) ST289 largely absent elsewhere such as in the USA [33], serotype 23F (SC8) largely ST802 and 6A (multiple STs and SCs) were also common in Malawi particularly in the under-five aged children. Although PMEN clones are globally prevalent [34], only three PMEN clones namely PMEN19 (SC1), PMEN25 (SC12) and PMEN27 (SC2) were identified in Malawi consistent with previous data [16]. While both PMEN19 and PMEN27 were associated with serotypes 5 and 1 respectively as in other countries, PMEN25 isolates in Malawi were associated with only serotype 14 but elsewhere it has been associated with both serotypes 15A and 19A [35] suggesting the occurrence of ST63^15A^ → ST63^14^ capsule switch in Malawi.

It has been shown that there was a decrease in IPD incidence from six years prior to PCV13 implementation in Malawi in 2011 [3], [6], however, our findings suggest this was not associated with decrease in specific serotypes but possibly an increased population host immunity possibly due to the nationwide scale-up of antiretroviral therapy (ART), cotrimoxazole prophylaxis and food security as previously reported [6], [37]. While our dataset suggested a decrease in prevalence of serotype 1 during the study period, this may reflect sampling bias for serotype 1 isolates particularly between 2009 and 2010 due to sequencing of serotype 1 isolates for another study [36]. This was confirmed using by randomly serotyping isolates from the archive (unpublished data), which showed no decrease in prevalence of serotype 1 but an increase in serotype 5 in 2010 possibly because of a local outbreak, although our findings appeared to overestimate the increase of serotype 5 in 2010 due to under sampling of serotype 1. Coalescent analysis of serotype 1 isolates showed stable population sizes, which provides further evidence that serotype 1’s population did not change pre-vaccination. These findings show that the pneumococcal population structure was stable before vaccination despite the decrease in disease incidence, which was driven by increased population immunity rather than decrease of certain serotypes.

Emergence and clonal expansion of antibiotic resistant strains complicates treatment and increases likelihood for severe outcomes because of treatment failure [38]. Highest resistance rates were unexpectedly observed in serotype 1 (SC2), which is atypical and challenges conventional knowledge that rarely carried are usually associated with low antimicrobial resistance rates due to limited recombination [39]. The reported antibiotic resistance rates in Malawi are similar to those in other African settings [31], [40] but are higher than observed before vaccination in high income countries including USA with the exception of penicillin and macrolide (erythromycin), which are associated with higher resistance in the USA [41] but in Malawi are associated with very low resistance rates. The observation that highly resistant antibiotics (tetracycline, chloramphenicol and cotrimoxazole) were associated with higher resistance among VT than NVT isolates suggest that their resistance rates will decrease post-PCV13 implementation but penicillin resistance may increase slightly because of its higher prevalence among NVTs than VTs although this was not statistically significant. An increase of NVT serotypes post-vaccination such as 12F (SC10), which showed the second highest MDR rate (57.9%) in Malawi after serotype 1 (81.9%) remain a significant concern. Serotype 12F exhibits high attack rate [42] and has been associated with outbreaks globally [43], [44]. Together these data suggest that while implementation of PCV13 will reduce the disease burden and antimicrobial resistance rates but continued surveillance to monitor potential replacement serotypes such as serotype 12F to remain crucial.

Capsule switching occurs predominantly due to recombination and it leads to the emergence of vaccine escape serotypes [28], [45]. Such capsule switching is more likely to occur in settings with high levels of recombination such as in Malawi [22] where it may promote the emergence of vaccine-escape serotypes. Our study showed high occurrence of capsule-switching across the phylogeny and in some of the lineages the capsule-switch variants were highly successful and subsequently replaced the original serotype as clearly depicted by the existence of multiple lineages of same serotype such as serotype 6A in SC3, SC15 and SC21. However, with regards to serotype replacement, most capsule-switch variants with an NVT capsule were of low prevalence than VT-associated capsule-switch variants suggesting that majority of the pre-existing capsule-switched variants will be cleared post-vaccination. Further analysis showed recent emergence of the pneumococcal lineages in Malawi, which suggests recent importation or clonal expansion of certain sub-clades.

Several limitations need to be acknowledged. Firstly, we did not perform temporal analysis of the serotypes because of potential under sampling of serotype 1 isolates between 2008 and 2010 but this did not affect our estimates of serotype prevalence because we did pooled analysis of the samples. Secondly, we did not include carriage samples in our analysis, which would have revealed additional serotypes not common in IPD but cause carriage in Malawi. Thirdly, due to fewer number of isolates sampled in certain years, our dataset was not equipped to accurately show changes in the prevalence of serotypes over time.

In conclusion, this study shows a high genetic diversity and stability of pneumococcal lineages and serotypes before the implementation of PCV13 vaccine in 2011. Serotype 1 accounted for majority of the IPD cases but the observation of highest resistance rates in this serotype defies conventional knowledge that infrequently carried pneumococcal lineages are typically susceptible to antibiotics due to limited recombination. While occurrence of natural capsule switching was evident, our findings suggest that serotype replacement post-PCV13 implementation is likely to be due to clonal expansion of NVT lineages rather than pre-existing capsule switched serotypes because highly prevalent acquired capsule-types were commonly associated with acquisition of vaccine-targeted capsules. The recent emergence of pneumococcal lineages and serotypes, and the potential emergence of replacement serotypes post-vaccination shows that continued surveillance is crucial to understand the pneumococcal epidemiology and to inform infection prevention and control strategies. The baseline genomic data provided in this study will enable more accurate analysis of the lineage-specific changes in serotype distribution post-PCV13 implementation in Malawi.

## Author’s contribution

CC, SDB, WPH and DBE conceived the study. SDB, WPH and DBE supervised study. NF, RSH and DBE provided clinical samples. MA, JEC, AWK and CP did serotyping and DNA extraction. SDB and RAG did whole genome sequencing. CC, SDB, WPH and DBE analyzed the data. CC, WPH, SDB and DBE wrote the manuscript. CPA, RAG, JEC, AWK, NB, AMK, CLM, AK, LM, RFB, NF, RSH, WPH, SDB and DBE reviewed the manuscript. All the authors have read and approved the final manuscript.

## Funding sources

This work was supported by the Bill and Melinda Gates Foundation, Wellcome Trust – United Kingdom awards number OPP1023440 (DBE and SDB) and OPP1034556 (RFB, SDB and LM). Activities at the Malawi-Liverpool-Wellcome Trust Clinical Research Programme were supported by a core award number 084679/Z/08/Z from the Wellcome Trust. CC acknowledges PhD Studentship funding from the Commonwealth Scholarship Commission in the UK. The content is solely the responsibility of the authors and does not necessarily represent the official views of the funding agencies and the Centers for Disease Control and Prevention (CDC).
