# Supplementary material for: Population genetic structure, antibiotic resistance, capsule switching and evolution of invasive pneumococci before conjugate vaccination in Malawi
Source: Vaccine. 2017 Aug 16;35(35Part B):4594–602. doi: 10.1016/j.vaccine.2017.07.009 (PMC5571440; doi:10.1016/j.vaccine.2017.07.009)
Supplement: Supplementary data 1 [file mmc1.docx]

**SUPPLEMENTAL MATERIAL**

**Population genetic structure, antibiotic resistance, capsule switching and evolution of invasive pneumococci before conjugate vaccination in Malawi**

Chrispin Chaguza^a,b^, Jennifer E. Cornick^a,b^, Cheryl P. Andam^a,c,^*, Rebecca A. Gladstone^d^, Maaike Alaerts^a,b,‡^, Patrick Musicha^a,b,e^, Chikondi Peno^b^, Naor Bar-Zeev^a,b^, Arox W. Kamng’ona^a,b,e^, Anmol M. Kiran^a,b^, Chisomo L. Msefula^b,e^, Lesley McGee^f^, Robert F. Breiman^g^, Aras Kadioglu^a^, Neil French^a,b^, Robert S. Heyderman^b,h^, William P. Hanage^c,†^, Stephen D. Bentley^a,d,†^ and Dean B. Everett^a,b,†,§^

^a^Department of Clinical Infection, Microbiology and Immunology, Institute of Infection and Global Health, University of Liverpool, Liverpool, UK

^b^Malawi-Liverpool-Wellcome Trust Clinical Research Programme, Blantyre, Malawi

^c^Center for Communicable Disease Dynamics, Department of Epidemiology, Harvard T.H. Chan School of Public Health, Boston, Massachusetts, USA

^d^Pathogen Genomics, Wellcome Trust Sanger Institute, Hinxton, Cambridge, UK

^e^Department of Biomedical Sciences, University of Malawi College of Medicine, Blantyre, Malawi

^f^Respiratory Diseases Branch, Centers for Disease Control and Prevention, Atlanta, USA

^g^Hubert Department of Global Health, Rollins School of Public Health, Emory University, Atlanta, USA

^h^Division of Infection and Immunity, University College London, London, UK

**Running title**: Pneumococcal genomic epidemiology in Malawi

^§^**Corresponding author**

Dr. Dean B. Everett BSc, DLSHTM, MSc, PhD

Reader in Molecular Microbiology

Faculty of Health and Life Sciences

Department of Clinical Infection, Microbiology and Immunology

Institute of Infection and Global Health, University of Liverpool

8 West Derby Street, Liverpool, L69 7BE, UK

Dean.Everett@liverpool.ac.uk

^†^These senior authors contributed equally to this article.

**Current affiliations:**

*Department of Molecular, Cellular and Biomedical Sciences, University of New Hampshire, Durham, USA

^‡^Department of Medical Genetics, University of Antwerp, Antwerp, Belgium

**MATERIALS AND METHODS**

**Sampling strategy**

A convenient sample of 585 invasive pneumococcal isolates was retrospectively sampled for whole genome sequencing. The isolates were obtained from the bacterial strain archive at the Malawi-Liverpool-Wellcome Trust Clinical Research Programme (www.mlw.medcol.mw) (**Supplemental Table S2**). The isolates were collected at Queen Elizabeth Central Hospital (QECH), Blantyre, Malawi, from adult and pediatric patients with IPD before (2004-2010) PCV13 vaccine introduction in 2011. The isolates were obtained from blood and cerebral spinal fluid (CSF) and constituted 65.5% and 38.5% of the samples respectively. Of these, 40.9% were obtained from females, 41.0% from males while other isolates had unknown gender. The isolates were collected from 2004 (n=92), 2005 (n=131), 2006 (n=94), 2007 (n=52), 2008 (n=77), 2009 (n=59) and 2010 (n=80). The minimum incidence for pneumococci during the sampling period was estimated from the total number of blood and CSF cultures from the strain archive.

**Culturing of pneumococcal isolates**

Pneumococcal isolates were stored in Microbank bacterial preservers (Prolab Diagnostics, ON, Canada) in freezers at -80°C. The isolates were then streaked onto 5% sheep’s blood agar supplemented with 5μg/μl gentamicin (SBG) plates. Optochin disc was inserted between the primary and secondary streaks and incubated at 37°C for 18 to 24 hours. Following the bacterial growth on the primary plate, we subcultured a single pneumococcal colony onto a fresh SBG plate with Optochin disc and incubated at 37ºC in 5% CO_2_ for 18 to 24 hours. Serotyping was done using were PCR [26] and confirmed using genomic data [27]. Sequence typing was done using multilocus sequence typing (MLST) scheme for the pneumococcus species [28, 29].

**Pretreatment of pneumococci and DNA extraction**

To extract pneumococcal DNA, we harvested the pneumococcal cells and re-suspended them in 180μl enzymatic lysis buffer made from 20mM Tris-Cl (Life Technologies, CA, USA), pH 8.0, 2mM sodium EDTA (Promega, WI, USA), 1.2% Triton X-100 (Thermo Scientific, MA, USA) lysozyme from chicken egg, 20mg/ml (Sigma-Aldrich, MO, USA) and incubated them at 37°C for 1 hour. Following this, we centrifuged the samples at 7500rpm for 10 minutes and then re-suspended the bacterial pellet in 15μl proteinase K (Qiagen, Hilden, Germany), 85μl buffer AL (Qiagen, Hilden, Germany) and incubated them at 56°C for 30 minutes. We then added 200μl of buffer AL and mixed the sample thoroughly by vortexing to 300μl the pretreated sample. DNA extraction from the pretreated sample was done using the QIAamp DNA mini kit (Qiagen, Hilden, Germany), QIAgen Biorobot (Qiagen, Hilden, Germany) or the Wizard® DNA Genomic DNA Purification Kit (Promega, WI, USA) following manufacturer’s instructions. We quantified the extracted DNA using the dsDNA Broad Range Assay on Qubit® 2.0 Fluorometer (Thermo Scientific, MA, USA).

**Whole genome sequencing**

Genomic DNA libraries were prepared and sequenced at the Wellcome Trust Sanger Institute using Illumina Genome Analyzer II and HiSeq platform (Illumina, CA, USA). All the generated sequence reads were deposited in the European Nucleotide Archive (ENA) under accession numbers in supplemental table S2. The sequence assemblies were generated using a pipeline [1] that uses Velvet v1.2.09 [2] and velvetOptimiser v2.2.5 [3] which used k-mers that ranged from 66% to 90% of the read length, SSPACE Basic v2.0 [4] to scaffold the assembly using 16 iterations, GapFiller v1.10 [5] to fill gaps in the assembly and finally re-mapping the reads to the assembly using SMALT v0.7.4 (<https://sourceforge.net/projects/smalt/>). The mean genome size for the generated assemblies was 2103839bp and ranged from 1911645bp to 2270200bp, the mean contig length was 41835bp and ranged from 11052bp to 135586bp and the mean number of contigs was 60.28 and ranged from 16 to 198 contigs. The mean N50 value for the assemblies was 90538bp and ranged from 24962bp to 399760bp.

**Phenotypic and genomic determination of antibiotic susceptibility profiles**

Antimicrobial sensitivity testing was done using the disc diffusion method. Several pneumococcal colonies were inoculated on Iso-sensitest agar (Oxoid, UK) with 5% sheep blood and 20mg/L β-nicotinamide adenine dinucleotide (ISB) plate and spread across the plate using a wet sterile cotton tipped stick. A disc dispenser (Oxoid, UK) containing oxacillin 1μg, chloramphecol 10μg, cotrimoxazole 25μg, erythromycin 5μg and tetracycline 10μg discs was placed on the plate and incubated at 37°C with 5% CO_2_ for 18 to 24 hours. Zone diameters were measured and interpreted using the British Society Antimicrobial Chemotherapy (BSAC) guidelines.

Antibiotic resistance conferring genes and conjugative elements were screened in the genomes using nucleotide BLAST v2.2.30 [6]. We set an E-value cutoff lower than 0.001 for the highest scoring pair (HSP) matches from BLAST to consider them non-spurious hits. We combined the antibiotic resistance-conferring element ‘present’ in the genomes when there was at least 80% query sequence coverage and average nucleotide sequence identity.

**Genomic and phylogenetic analysis**

We identified the core and accessory genes by clustering the coding sequences identified in each isolate using CD-HIT v4.6 [7] within the Roary v3.6.1 bacterial analysis pipeline [8]. The distribution of the core and accessory genes were summarized (Supplemental Fig. S2) and each core gene was aligned separately using MAFFT v7.025 [9] and then concatenated with other aligned core genes to generate a full core genome alignment. Snp-Sites v2.3.2 [10] was used to identify single nucleotide polymorphisms (SNPs) from the 1.04Mb alignment of 1506 core genes and 67932 SNP sites were identified. From the core SNP alignment, we identified the pneumococcal population structure in terms of sequence clusters (SCs) that comprised of closely related isolates using the hierBAPS method in BAPS v6.0 [11, 12].

**Phylogenetic construction and recombination detection**

Maximum likelihood phylogenies were constructed from the core SNP alignment using FastTree SSE3 v2.1.3 [13] and RAxML v7.0.4 [14]. We ran FastTree and RAxML with a generalized time reversible (GTR) model [15], the Gamma (Υ) heterogeneity between nucleotide sites and 100 bootstrap replicates. We included a single classical non-typeable (NT) pneumococci from carriage for outgroup rooting of the phylogeny because it forms a genetically distinct lineage. Visualizations of each isolate’s metadata overlaid on the phylogenies were done using iToL v2.1 [16]. We also mapped the sequence reads for isolates using an SC-specific reference sequence using SMALT v0.5.8 in order to gain full alignments of each SC. Using these alignments, we then ran Gubbins v1.1.1 [17] to detect and remove sites with evidence of horizontal acquisition through recombination and used them for subsequent coalescent analysis.

**Dating times of emergence and evolution rates for lineages**

Using the generated recombination-free alignments for each SC, we constructed SC-specific phylogenies, which were used together with the alignments to determine the emergence times and evolution rates of the SCs. We checked for evidence of molecular clock (linear time dependent) evolution for each SC using TempEst (formerly known as Path-O-gen) [18]. The input files for BEAST v1.8 were generated using BEAUti v1.7.5 [19]. The estimates for the mean and highest posterior density (HPD) - a Bayesian equivalent of the confidence intervals (CI) – for the nucleotide substitution (mutation) rates and the time of emergence for the SCs with molecular clock-like evolution were summarized using Tracer v1.6. The coalescent softwares used were available from http://tree.bio.ed.ac.uk.

**Statistical analysis**

We used the student’s t-test with a Yates continuity correction to compare differences between proportions. Association between in vitro and genomically inferred antibiotic resistance phenotypes were assessed using Chi-squared test. Trends in prevalence of serotypes and antibiotic resistance were determined using Chi-squared test for trends in proportions. The statistical tests and generation of graphs was done in R v3.1.2 (R Core Team, 2014) and GraphPad Prism v7.0 ([www.graphpad.com](http://www.graphpad.com)).

**REFERENCES**

[1] Page AJ, De Silva N, Hunt M, Quail MA, Parkhill J, Harris SR, et al. Robust high throughput prokaryote de novo assembly and improvement pipeline for Illumina data. bioRxiv. 2016.

[2] Zerbino D, Birney E. Velvet: Algorithms for de novo short read assembly using de Bruijn graphs. Genome Res. 2008;18:821 - 9.

[3] Zerbino DR. Using the Velvet de novo assembler for short-read sequencing technologies. Current protocols in bioinformatics / editoral board, Andreas D Baxevanis [et al]. 2010;Chapter 11:Unit 11 5.

[4] Boetzer M, Henkel CV, Jansen HJ, Butler D, Pirovano W. Scaffolding pre-assembled contigs using SSPACE. Bioinformatics. 2011;27:578-9.

[5] Nadalin F, Vezzi F, Policriti A. GapFiller: a de novo assembly approach to fill the gap within paired reads. BMC Bioinformatics. 2012;13:S8.

[6] Altschul S, Madden T, Schaffer A, Zhang J, Zhang Z, Miller W, et al. Gapped BLAST and PSI-BLAST: a new generation of protein database search programs. Nucleic Acids Res. 1997;25:3389 - 402.

[7] Li W, Godzik A. Cd-hit: a fast program for clustering and comparing large sets of protein or nucleotide sequences. Bioinformatics. 2006;22:1658-9.

[8] Page AJ, Cummins CA, Hunt M, Wong VK, Reuter S, Holden MT, et al. Roary: rapid large-scale prokaryote pan genome analysis. Bioinformatics. 2015;31:3691-3.

[9] Katoh K, Standley DM. MAFFT multiple sequence alignment software version 7: improvements in performance and usability. Mol Biol Evol. 2013;30.

[10] Page AJ, Taylor B, Delaney AJ, Soares J, Seemann T, Keane JA, et al. SNP-sites: rapid efficient extraction of SNPs from multi-FASTA alignments. Microbial Genomics. 2016;2.

[11] Cheng L, Connor TR, Sirén J, Aanensen DM, Corander J. Hierarchical and spatially explicit clustering of DNA sequences with BAPS software. Molecular Biology and Evolution. 2013.

[12] Corander J, Waldmann P, Sillanpaa MJ. Bayesian analysis of genetic differentiation between populations. Genetics. 2003;163:367-74.

[13] Price MN, Dehal PS, Arkin AP. FastTree: Computing Large Minimum Evolution Trees with Profiles instead of a Distance Matrix. Molecular Biology and Evolution. 2009;26:1641-50.

[14] Stamatakis A. RAxML-VI-HPC: maximum likelihood-based phylogenetic analyses with thousands of taxa and mixed models. Bioinformatics. 2006;22:2688 - 90.

[15] Tavaré S. Some Probabilistic and Statistical Problems in the Analysis of DNA Sequences. American Mathematical Society: Lectures on Mathematics in the Life Sciences: Amer Mathematical Society; 1986. p. 57-86.

[16] Letunic I, Bork P. Interactive Tree Of Life v2: online annotation and display of phylogenetic trees made easy. Nucleic Acids Research. 2011;39:W475-W8.

[17] Croucher NJ, Page AJ, Connor TR, Delaney AJ, Keane JA, Bentley SD, et al. Rapid phylogenetic analysis of large samples of recombinant bacterial whole genome sequences using Gubbins. Nucleic Acids Research. 2014.

[18] Rambaut A, Lam TT, Max Carvalho L, Pybus OG. Exploring the temporal structure of heterochronous sequences using TempEst (formerly Path-O-Gen). Virus evolution. 2016;2:vew007.

[19] Drummond AJ, Suchard MA, Xie D, Rambaut A. Bayesian phylogenetics with BEAUti and the BEAST 1.7. Mol Biol Evol. 2012;29:1969-73.

**TABLE AND FIGURE LEGENDS**

**Table S1. Pneumococcal isolates used in the present study (At the end).**

**Fig. S1. Distribution of pneumococcal isolates by age groups.** **(A)** Number of isolates by age groups. **(B)** Number of the invasive isolates by 5-year age groups. **(C)** Proportion of vaccine type (VT) and non-vaccine type (NVT) by age groups.

**Fig. S2. Temporal prevalence of pneumococcal serotypes in invasive disease.**

**Fig. S3.** Molecular clock signal in sequence clusters (SCs). Linear regression of the phylogenetic root-to-tip distances (total accumulated SNPs) and years of isolation. Plots marked with an asterisk (*) showed a significant relationship (molecular clock signal). SCs with significant relationships were used for coalescent analysis in BEAST.

**Fig. S4.** Mutation rates of the SCs with detectable molecular clock signal from BEAST.

**Fig. S5.** Acquisition and distribution of capsular types across the pneumococcal phylogeny in Malawi. All the capsule types associated with multiple phylogenetic lineages or SCs are shown. Presence of the capsule on each branch is shown in red.

**Table S1.** Pneumococcal isolates used in the present study.

| **Sample ID** | **Sequence Cluster (SC)** | **Year** | **Source** | **MLST** | **Serotype** | **ENA Accession** | **Gender** | **Age (Yrs)** |
| --- | --- | --- | --- | --- | --- | --- | --- | --- |
| C9801 | SC1 | 2004 | CSF | 289 | 5 | ERS553382 | Male |  |
| D27816 | SC1 | 2004 | Blood | 289 | 5 | ERS006730 | Female | 9 |
| A29101 | SC1 | 2004 | Blood | 289 | 5 | ERS012095 | Male |  |
| B9517 | SC1 | 2004 | CSF | 289 | 5 | ERS050449 | Male |  |
| A28426 | SC1 | 2004 | Blood | 289 | 5 | ERS006779 | Male | 36 |
| D33008 | SC1 | 2005 | Blood | 289 | 5 | ERS553572 | Male | 1 |
| A37482 | SC1 | 2005 | Blood | 289 | 5 | ERS050366 | Male | 45 |
| A38608 | SC1 | 2005 | Blood | 289 | 5 | ERS050369 | Female | 25 |
| C15256 | SC1 | 2006 | CSF | 289 | 5 | ERS006817 | Female |  |
| D40410 | SC1 | 2006 | Blood | 289 | 5 | ERS553403 | Male | 4 |
| C15085 | SC1 | 2006 | CSF | 289 | 5 | ERS553491 |  | 2 |
| C15085 | SC1 | 2006 | CSF | 289 | 5 | ERS006744 |  | 2 |
| A43327 | SC1 | 2006 | Blood | 289 | 5 | ERS006785 |  |  |
| A43874 | SC1 | 2006 | Blood | 289 | 5 | ERS006793 |  |  |
| D40273 | SC1 | 2006 | Blood | 289 | 5 | ERS026051 | Male |  |
| A43030 | SC1 | 2006 | Blood | 289 | 5 | ERS050375 |  |  |
| A43904 | SC1 | 2006 | Blood | 289 | 5 | ERS050378 |  |  |
| A44175 | SC1 | 2006 | Blood | 289 | 5 | ERS050383 |  |  |
| C15358 | SC1 | 2006 | CSF | 289 | 5 | ERS214955 | Female | 4 |
| C17600 | SC1 | 2007 | CSF | 289 | 5 | ERS625415 | Male |  |
| C16023 | SC1 | 2007 | CSF | 289 | 5 | ERS024523 |  | 1 |
| D44249 | SC1 | 2007 | Blood | 289 | 5 | ERS553383 |  | 3 |
| C16309 | SC1 | 2007 | CSF | 289 | 5 | ERS553421 | Female |  |
| C17208 | SC1 | 2007 | CSF | 289 | 5 | ERS553509 | Male |  |
| B16728 | SC1 | 2007 | CSF | 289 | 5 | ERS003576 | Female | 31 |
| D46845 | SC1 | 2008 | Blood | 289 | 5 | ERS050639 |  | 6 |
| A52248 | SC1 | 2008 | Blood | 245 | 5 | ERS050393 | Female | 20 |
| D48490 | SC1 | 2008 | Blood | 289 | 5 | ERS050641 | Female | 12 |
| D49319 | SC1 | 2008 | Blood | 289 | 5 | ERS050642 | Female |  |
| C19309 | SC1 | 2008 | CSF | 289 | 5 | ERS214935 | Male | 4 |
| A55299 | SC1 | 2009 | Blood | 289 | 5 | ERS553495 | Female | 35 |
| A57058 | SC1 | 2009 | Blood | 289 | 5 | ERS625696 | Female | 41 |
| D53800 | SC1 | 2009 | Blood | 289 | 5 | ERS625949 | Male |  |
| D52077 | SC1 | 2009 | Blood | 289 | 5 | ERS050644 | Male | 6 |
| A59588 | SC1 | 2010 | Blood | 289 | 5 | ERS553385 | Male | 28 |
| A59699 | SC1 | 2010 | Blood | 289 | 5 | ERS553396 | Female | 26 |
| A59303 | SC1 | 2010 | Blood | 289 | 5 | ERS553378 | Female | 27 |
| D54465 | SC1 | 2010 | Blood | 289 | 5 | ERS625573 |  |  |
| 1004982 | SC1 | 2010 | Blood | 289 | 5 | ERS625692 | Male | 1 |
| A59302 | SC1 | 2010 | Blood | 289 | 5 | ERS625721 | Female | 26 |
| A59399 | SC1 | 2010 | Blood | 289 | 5 | ERS625732 | Male |  |
| D54555 | SC1 | 2010 | Blood | 289 | 5 | ERS625937 | Female | 6 |
| 1003391 | SC1 | 2010 | Blood | 289 | 5 | ERS625729 | Male | 7 |
| B21609 | SC1 | 2010 | CSF | 289 | 5 | ERS050439 | Female | 17 |
| A59399 | SC1 | 2010 | Blood | 289 | 5 | ERS050407 | Male | 31 |
| D54582 | SC1 | 2010 | Blood | 289 | 5 | ERS214920 | Female | 2 |
| C23870 | SC1 | 2010 | CSF | 289 | 5 | ERS214923 | Female | 1 |
| D57096 | SC1 | 2010 | Blood | 289 | 5 | ERS214926 | Male | 1 |
| 1007374 | SC1 | 2010 | Blood | 289 | 5 | ERS214910 |  | 1 |
| D56382 | SC1 | 2010 | Blood | 289 | 5 | ERS214933 | Female | 1 |
| 1005787 | SC1 | 2010 | Blood | 289 | 5 | ERS214944 |  | 1 |
| C22318 | SC1 | 2010 | CSF | 289 | 5 | ERS214914 | Male | 1 |
| 1005015 | SC1 | 2010 | Blood | 289 | 5 | ERS214994 |  | 8 |
| A59375 | SC1 | 2010 | Blood | 289 | 5 | ERS215012 | Male | 31 |
| D27639 | SC2 | 2004 | Blood | 217 | 1 | ERS399067 | Male |  |
| A30425 | SC2 | 2004 | Blood | 217 | 1 | ERS553402 | Female |  |
| D28038 | SC2 | 2004 | Blood | 217 | 1 | ERS553567 | Male |  |
| D25066 | SC2 | 2004 | Blood | 217 | 1 | ERS553571 | Female |  |
| D25796 | SC2 | 2004 | Blood | 217 | 1 | ERS024510 | Female | 8 |
| D28981 | SC2 | 2004 | Blood | 217 | 1 | ERS024516 |  | 3 |
| A28816 | SC2 | 2004 | Blood | 217 | 1 | ERS012093 | Male |  |
| A31105 | SC2 | 2004 | Blood | 217 | 1 | ERS050335 | Male | 36 |
| A31584 | SC2 | 2004 | Blood | 217 | 1 | ERS050342 | Female | 30 |
| D26805 | SC2 | 2004 | Blood | 217 | 1 | ERS214925 | Female |  |
| D25748 | SC2 | 2004 | Blood | 217 | 1 | ERS214928 | Male |  |
| D25235 | SC2 | 2004 | Blood | 217 | 1 | ERS214981 |  |  |
| C9938 | SC2 | 2004 | CSF | 217 | 1 | ERS398799 |  |  |
| D27106 | SC2 | 2004 | Blood | 217 | 1 | ERS399226 |  |  |
| C9471 | SC2 | 2004 | CSF | 217 | 1 | ERS006723 |  |  |
| A30928 | SC2 | 2004 | Blood | 217 | 1 | ERS006788 | Male |  |
| A30338 | SC2 | 2004 | Blood | 217 | 1 | ERS006789 | Male |  |
| A32572 | SC2 | 2004 | Blood | 217 | 1 | ERS050346 | Female | 25 |
| D27102 | SC2 | 2004 | Blood | 11745 | 1 | ERS050494 | Female | 7 |
| D27175 | SC2 | 2004 | Blood | 11745 | 1 | ERS214985 | Female |  |
| D27540 | SC2 | 2004 | Blood | 11745 | 1 | ERS215001 |  |  |
| D33293 | SC2 | 2005 | Blood | 217 | 1 | ERS399068 | Male | 8 |
| C13208 | SC2 | 2005 | CSF | 217 | 1 | ERS399050 | Male | 8 |
| D31094 | SC2 | 2005 | Blood | 217 | 1 | ERS553381 |  | 1 |
| D32648 | SC2 | 2005 | Blood | 11745 | 1 | ERS553393 |  | 1 |
| C11657 | SC2 | 2005 | CSF | 217 | 1 | ERS553398 |  | 1 |
| D33521 | SC2 | 2005 | Blood | 217 | 1 | ERS006728 | Male | 3 |
| C13181 | SC2 | 2005 | CSF | 217 | 1 | ERS006746 | Female | 9 |
| C13215 | SC2 | 2005 | CSF | 217 | 1 | ERS006745 | Female | 11 |
| A37371 | SC2 | 2005 | Blood | 217 | 1 | ERS006772 | Female | 27 |
| A35070 | SC2 | 2005 | Blood | 217 | 1 | ERS006787 | Male | 21 |
| D34485 | SC2 | 2005 | Blood | 217 | 1 | ERS024512 | Female | 15 |
| A36633 | SC2 | 2005 | Blood | 217 | 1 | ERS006825 | Female | 31 |
| A34978 | SC2 | 2005 | Blood | 217 | 1 | ERS008321 |  | 42 |
| A37104 | SC2 | 2005 | Blood | 217 | 1 | ERS008326 | Male | 39 |
| B12423 | SC2 | 2005 | CSF | 217 | 1 | ERS008341 | Female | 39 |
| A33973 | SC2 | 2005 | Blood | 217 | 1 | ERS012120 | Female | 28 |
| A34030 | SC2 | 2005 | Blood | 217 | 1 | ERS012121 |  | 55 |
| A35279 | SC2 | 2005 | Blood | 217 | 1 | ERS012127 | Female | 34 |
| B11467 | SC2 | 2005 | CSF | 217 | 1 | ERS012133 | Female | 18 |
| B11505 | SC2 | 2005 | CSF | 217 | 1 | ERS012134 | Female | 26 |
| A35715 | SC2 | 2005 | Blood | 217 | 1 | ERS050350 | Male | 38 |
| A37046 | SC2 | 2005 | Blood | 217 | 1 | ERS050362 | Female | 24 |
| A37089 | SC2 | 2005 | Blood | 217 | 1 | ERS050363 | Female | 47 |
| B12027 | SC2 | 2005 | CSF | 217 | 1 | ERS050413 | Male | 20 |
| D35438 | SC2 | 2005 | Blood | 217 | 1 | ERS050496 | Female | 9 |
| C12085 | SC2 | 2005 | CSF | 217 | 1 | ERS553372 |  | 5 |
| C11020 | SC2 | 2005 | CSF | 303 | 1 | ERS006824 |  |  |
| A33056 | SC2 | 2005 | Blood | 303 | 1 | ERS003550 | Male | 28 |
| A35711 | SC2 | 2005 | Blood | 11745 | 1 | ERS008323 | Female | 52 |
| A34045 | SC2 | 2005 | Blood | 11745 | 1 | ERS012122 | Female | 63 |
| A36780 | SC2 | 2005 | Blood | 11745 | 1 | ERS012129 | Male | 23 |
| A37004 | SC2 | 2005 | Blood | 217 | 1 | ERS050361 | Female | 28 |
| C12797a | SC2 | 2005 | CSF | 217 | 1 | ERS050453 | Female | 5 |
| C14099 | SC2 | 2006 | CSF | 217 | 1 | ERS006734 | Female | 12 |
| A41093 | SC2 | 2006 | Blood | 11745 | 1 | ERS006774 |  |  |
| D38643 | SC2 | 2006 | Blood | 217 | 1 | ERS026042 | Female |  |
| A39247 | SC2 | 2006 | Blood | 217 | 1 | ERS012061 |  |  |
| A41267 | SC2 | 2006 | Blood | 217 | 1 | ERS012063 |  |  |
| A42174 | SC2 | 2006 | Blood | 217 | 1 | ERS012069 |  |  |
| A43126 | SC2 | 2006 | Blood | 217 | 1 | ERS050376 |  |  |
| A44013 | SC2 | 2006 | Blood | 217 | 1 | ERS050379 |  |  |
| A44026 | SC2 | 2006 | Blood | 217 | 1 | ERS050380 |  |  |
| A44049 | SC2 | 2006 | Blood | 217 | 1 | ERS050381 |  |  |
| D38891 | SC2 | 2006 | Blood | 217 | 1 | ERS553561 | Female | 4 |
| C14940 | SC2 | 2006 | CSF | 11745 | 1 | ERS006731 | Male | 6 |
| A41626 | SC2 | 2006 | Blood | 217 | 1 | ERS006783 |  |  |
| C14249 | SC2 | 2006 | CSF | 217 | 1 | ERS006820 | Male |  |
| B14935 | SC2 | 2006 | CSF | 217 | 1 | ERS012073 | Male | 21 |
| A44163 | SC2 | 2006 | Blood | 217 | 1 | ERS050382 |  |  |
| D44741 | SC2 | 2007 | Blood | 217 | 1 | ERS553377 | Female | 1 |
| A47864 | SC2 | 2007 | Blood | 217 | 1 | ERS553455 | Male | 37 |
| C16000 | SC2 | 2007 | CSF | 217 | 1 | ERS006743 | Male |  |
| A46444 | SC2 | 2007 | Blood | 217 | 1 | ERS006799 | Male | 30 |
| B17099 | SC2 | 2007 | CSF | 217 | 1 | ERS003569 | Male |  |
| A49293 | SC2 | 2007 | Blood | 217 | 1 | ERS003573 | Male | 45 |
| C15514 | SC2 | 2007 | CSF | 217 | 1 | ERS008357 | Male | 5 |
| D40696 | SC2 | 2007 | Blood | 217 | 1 | ERS008363 | Male | 5 |
| B15976 | SC2 | 2007 | CSF | 217 | 1 | ERS012084 | Male | 34 |
| D44689 | SC2 | 2007 | Blood | 217 | 1 | ERS214939 | Female | 3 |
| D40872 | SC2 | 2007 | Blood | 217 | 1 | ERS215010 | Male | 1 |
| D42785 | SC2 | 2007 | Blood | 11745 | 1 | ERS553497 | Female | 10 |
| D42299 | SC2 | 2007 | Blood | 217 | 1 | ERS026060 | Male | 1 |
| B16942 | SC2 | 2007 | CSF | 217 | 1 | ERS003577 | Male | 20 |
| B17224 | SC2 | 2007 | CSF | 217 | 1 | ERS050414 | Female | 70 |
| B17447 | SC2 | 2008 | CSF | 217 | 1 | ERS006803 | Male | 25 |
| C18031 | SC2 | 2008 | CSF | 217 | 1 | ERS026063 | Male | 4 |
| D48309 | SC2 | 2008 | Blood | 217 | 1 | ERS024531 | Female | 4 |
| C19609 | SC2 | 2008 | CSF | 217 | 1 | ERS006814 | Male | 1 |
| B17333 | SC2 | 2008 | CSF | 217 | 1 | ERS012088 | Male | 83 |
| A51425 | SC2 | 2008 | Blood | 217 | 1 | ERS050388 | Female | 25 |
| B17789 | SC2 | 2008 | CSF | 217 | 1 | ERS050415 | Male |  |
| B18042 | SC2 | 2008 | CSF | 217 | 1 | ERS050417 | Female | 17 |
| C19783 | SC2 | 2008 | CSF | 217 | 1 | ERS553493 | Female | 15 |
| B18718 | SC2 | 2008 | CSF | 303 | 1 | ERS003586 | Female | 23 |
| A53108 | SC2 | 2008 | Blood | 217 | 1 | ERS050395 | Male | 43 |
| A53532 | SC2 | 2008 | Blood | 11745 | 1 | ERS050397 | Male |  |
| D45902 | SC2 | 2008 | Blood | 217 | 1 | ERS050638 | Female | 9 |
| B17998 | SC2 | 2008 | CSF | 217 | 1 | ERS050416 | Male | 35 |
| A55084 | SC2 | 2009 | Blood | 217 | 1 | ERS006809 | Female | 43 |
| A55581 | SC2 | 2009 | Blood | 217 | 1 | ERS398974 | Female | 29 |
| C21127 | SC2 | 2009 | CSF | 11745 | 1 | ERS050480 | Male | 8 |
| A54136 | SC2 | 2009 | Blood | 217 | 1 | ERS214980 | Male | 32 |
| A55122 | SC2 | 2009 | Blood | 217 | 1 | ERS006811 | Male | 37 |
| A59803 | SC2 | 2010 | Blood | 217 | 1 | ERS553503 | Male | 20 |
| 1005628 | SC2 | 2010 | Blood | 217 | 1 | ERS625716 | Female | 35 |
| 1003235 | SC2 | 2010 | CSF | 217 | 1 | ERS625726 | Female | 1 |
| 1004168 | SC2 | 2010 | Blood | 217 | 1 | ERS214960 |  | 7 |
| C22697 | SC2 | 2010 | CSF | 217 | 1 | ERS553389 | Female |  |
| C22964 | SC2 | 2010 | CSF | 217 | 1 | ERS625722 | Female | 9 |
| A58264 | SC2 | 2010 | Blood | 217 | 1 | ERS050405 | Female | 33 |
| A59539 | SC2 | 2010 | Blood | ST217LV | 1 | ERS050408 | Male | 24 |
| 1007130 | SC2 | 2010 | Blood | 9529 | 1 | ERS214927 |  | 3 |
| C12270 | SC3 | 2005 | CSF | 9465 | 6A | ERS215006 | Female | 1 |
| D38094 | SC3 | 2006 | Blood | 2902 | 6A | ERS006733 | Male |  |
| D38023 | SC3 | 2006 | Blood | 9466 | 6A | ERS012079 | Male | 1 |
| B15901 | SC3 | 2007 | CSF | 2902 | 6A | ERS012083 | Female |  |
| A51131 | SC3 | 2008 | Blood | 2902 | 6A | ERS003578 | Male | 16 |
| A53886 | SC3 | 2009 | Blood | 2902 | 6A | ERS050399 | Male | 45 |
| A54130 | SC3 | 2009 | Blood | 2902 | 6A | ERS050400 | Male | 24 |
| C21075 | SC3 | 2009 | CSF | 2902 | 6A | ERS050478 | Male | 1 |
| B21980 | SC3 | 2010 | CSF | 2902 | 6A | ERS050441 | Male |  |
| C22644 | SC3 | 2010 | CSF | 2902 | 6A | ERS050481 | Female | 2 |
| C13355 | SC3 | 2005 | CSF | 2902 | 6B | ERS625385 | Female | 1 |
| C14798 | SC3 | 2006 | CSF | 2902 | 6B | ERS625386 | Male | 1 |
| D42617 | SC3 | 2007 | Blood | 2902 | 6B | ERS553374 | Female | 3 |
| D42617 | SC3 | 2007 | Blood | 2902 | 6B | ERS553374 | Female | 3 |
| D55333 | SC3 | 2010 | Blood | 2902 | 6B | ERS214930 | Female | 1 |
| D26855 | SC3 | 2004 | Blood | 9466 | 6C | ERS214921 |  |  |
| A31361 | SC4 | 2004 | Blood | 2062 | 19A | ERS050341 | Female |  |
| A31349 | SC4 | 2004 | Blood | 9457 | 19A | ERS050340 | Female | 54 |
| A36652 | SC4 | 2005 | Blood | 2062 | 19A | ERS012128 | Female | 29 |
| A37452 | SC4 | 2005 | Blood | 9457 | 19A | ERS050365 | Female | 30 |
| D32370 | SC4 | 2005 | Blood | 9457 | 19A | ERS214950 | Female | 3 |
| C12139 | SC4 | 2005 | CSF | 9457 | 19A | ERS214962 |  | 3 |
| A35432 | SC4 | 2005 | Blood | 9457 | 19A | ERS008322 | Female | 51 |
| B11684 | SC4 | 2005 | CSF | 9457 | 19A | ERS008337 | Male | 51 |
| A36173 | SC4 | 2005 | Blood | 9457 | 19A | ERS050355 | Male | 30 |
| A35786 | SC4 | 2005 | Blood | 2062 | 19A | ERS050352 | Female | 34 |
| A40931 | SC4 | 2006 | Blood | 7898LV | 19A | ERS008329 |  |  |
| C20833 | SC4 | 2009 | CSF | 9457 | 19A | ERS050474 | Male | 1 |
| D55280 | SC4 | 2010 | Blood | 2062 | 19A | ERS214941 | Female | 1 |
| A57454 | SC4 | 2010 | Blood | 9457 | 19A | ERS625758 | Male |  |
| A33075 | SC5 | 2005 | Blood | 705 | 16F | ERS012115 | Male | 28 |
| D30716 | SC5 | 2005 | Blood | 705 | 16F | ERS012142 |  | 3 |
| A35997 | SC5 | 2005 | Blood | 705 | 16F | ERS050353 | Female | 25 |
| A36147 | SC5 | 2005 | Blood | 705 | 16F | ERS050354 | Male | 38 |
| D37909 | SC5 | 2006 | Blood | 705 | 16F | ERS026050 | Female | 1 |
| A42190 | SC5 | 2006 | Blood | 705 | 16F | ERS012070 |  |  |
| A41312 | SC5 | 2006 | Blood | 705 | 16F | ERS012064 |  |  |
| D39297 | SC5 | 2006 | Blood | 705 | 16F | ERS214997 | Female | 1 |
| C17353 | SC5 | 2007 | CSF | 705 | 16F | ERS399189 | Female | 1 |
| D40857 | SC5 | 2007 | Blood | 705 | 16F | ERS026053 | Female | 9 |
| D42083 | SC5 | 2007 | Blood | 705 | 16F | ERS399190 | Male | 1 |
| A35264 | SC6 | 2005 | Blood | 700 | 3 | ERS050349 | Male | 32 |
| D36881 | SC6 | 2006 | Blood | 700 | 3 | ERS026041 | Male |  |
| A40924 | SC6 | 2006 | Blood | 700 | 3 | ERS050370 |  |  |
| A52402 | SC6 | 2008 | Blood | 700 | 3 | ERS050394 | Female | 20 |
| A56952 | SC6 | 2009 | Blood | 700 | 3 | ERS625714 | Female |  |
| B20539 | SC6 | 2009 | CSF | 700 | 3 | ERS050432 | Female | 28 |
| B20605 | SC6 | 2009 | CSF | 700 | 3 | ERS050433 | Male | 46 |
| A57264 | SC6 | 2009 | Blood | 700 | 3 | ERS214996 | Female | 36 |
| A60311 | SC6 | 2010 | Blood | 700 | 3 | ERS214953 | Male | 50 |
| D23908 | SC6 | 2004 | Blood | 230LV | 19A | ERS024514 |  |  |
| D49759 | SC6 | 2009 | Blood | 1611 | 19A | ERS050643 | Female | 1 |
| A29037 | SC7 | 2004 | Blood | 7055 | 10B | ERS012094 | Male | 31 |
| B9399 | SC7 | 2004 | CSF | 7055 | 10B | ERS012106 | Male |  |
| D31621 | SC7 | 2005 | Blood | 7055 | 10B | ERS006727 |  | 1 |
| C11367 | SC7 | 2005 | CSF | 7055 | 10B | ERS026044 |  | 7 |
| B11601 | SC7 | 2005 | CSF | 7055 | 10B | ERS003555 | Male |  |
| A34857 | SC7 | 2005 | Blood | 7055 | 10B | ERS050348 | Male | 28 |
| D30686 | SC7 | 2005 | Blood | 7055 | 10B | ERS214912 | Male | 4 |
| A42964 | SC7 | 2006 | Blood | 7055LV | 10B | ERS006806 |  |  |
| A42347 | SC7 | 2006 | Blood | 7055 | 10B | ERS008331 |  |  |
| B14457 | SC7 | 2006 | CSF | 7055 | 10B | ERS008345 | Male | 50 |
| B16827 | SC7 | 2007 | CSF | 7055 | 10B | ERS012087 | Male | 28 |
| A51944 | SC7 | 2008 | Blood | 7055 | 10B | ERS050390 | Male | 35 |
| A53112 | SC7 | 2008 | Blood | 7055 | 10B | ERS050396 | Female | 40 |
| B18107 | SC7 | 2008 | CSF | 7055 | 10B | ERS050418 | Female | 20 |
| A34568 | SC7 | 2005 | Blood | 4084 | 33D | ERS012125 | Female | 23 |
| C18320 | SC7 | 2008 | CSF | 4084 | 33D | ERS050462 | Female |  |
| D56471 | SC7 | 2010 | Blood | 7055 | 6A | ERS214917 | Male | 2 |
| D28525 | SC8 | 2004 | Blood | 802 | 23F | ERS553411 |  |  |
| A32273 | SC8 | 2004 | Blood | 802LV | 23F | ERS008320 | Female |  |
| B10366 | SC8 | 2004 | CSF | 802LV | 23F | ERS008335 | Male | 44 |
| C10263 | SC8 | 2004 | CSF | 9530 | 23F | ERS398852 |  |  |
| D26316 | SC8 | 2004 | Blood | 802 | 23F | ERS006738 | Male |  |
| C11481 | SC8 | 2005 | CSF | 802 | 23F | ERS553417 |  | 3 |
| D30625 | SC8 | 2005 | Blood | 802 | 23F | ERS012141 |  | 3 |
| D30625 | SC8 | 2005 | Blood | 802 | 23F | ERS214978 | Male | 3 |
| D34320 | SC8 | 2005 | Blood | 802 | 23F | ERS398973 | Male | 4 |
| C11283 | SC8 | 2005 | CSF | 802 | 23F | ERS553401 |  | 1 |
| A36756 | SC8 | 2005 | Blood | 802 | 23F | ERS003552 | Female | 40 |
| A33308 | SC8 | 2005 | Blood | 802 | 23F | ERS012116 | Female | 22 |
| A37297 | SC8 | 2005 | Blood | 802 | 23F | ERS050364 | Female | 36 |
| B11055 | SC8 | 2005 | CSF | 802LV | 23F | ERS050411 | Female |  |
| B14767 | SC8 | 2006 | CSF | 802LV | 23F | ERS012072 | Male |  |
| B15188 | SC8 | 2006 | CSF | 802 | 23F | ERS012075 | Female | 71 |
| C499 | SC8 | 2006 | CSF | 802 | 23F | ERS399007 |  | 1 |
| C13420 | SC8 | 2006 | CSF | 9530 | 23F | ERS399272 | Female | 1 |
| C14811 | SC8 | 2006 | CSF | 802 | 23F | ERS553442 |  | 1 |
| C15291 | SC8 | 2006 | CSF | 802 | 23F | ERS026058 | Female | 1 |
| D43782 | SC8 | 2007 | Blood | 9538 | 23F | ERS006758 | Female | 1 |
| D42660 | SC8 | 2007 | Blood | 802 | 23F | ERS026061 | Female | 3 |
| D46897 | SC8 | 2008 | Blood | 9535 | 23F | ERS024529 | Female |  |
| B18340 | SC8 | 2008 | CSF | 802 | 23F | ERS050420 | Male | 2 |
| B18678 | SC8 | 2008 | CSF | 802 | 23F | ERS050423 | Male | 31 |
| C17952 | SC8 | 2008 | CSF | 802 | 23F | ERS050459 | Female | 1 |
| C23361 | SC8 | 2010 | CSF | 802 | 23F | ERS214931 | Male | 2 |
| D56983 | SC8 | 2010 | Blood | 9530 | 23F | ERS398845 | Female | 1 |
| D57353 | SC8 | 2010 | Blood | 802 | 23F | ERS625932 | Female | 15 |
| 1003082 | SC8 | 2010 | Blood | 802 | 23F | ERS214940 |  | 1 |
| A36529 | SC9 | 2005 | Blood | 172 | 15B/C | ERS050356 | Female | 45 |
| D41212 | SC9 | 2007 | Blood | 172 | 15B/C | ERS398919 | Male | 2 |
| D41212 | SC9 | 2007 | Blood | 172 | 15B/C | ERS026059 | Female | 2 |
| A35792 | SC9 | 2005 | Blood | 1131LV | 19A | ERS008324 | Female | 55 |
| B11856 | SC9 | 2005 | CSF | 1131LV | 19A | ERS008339 | Male | 55 |
| D46120 | SC9 | 2008 | Blood | 10599 | 19A | ERS024528 | Male |  |
| D33693 | SC9 | 2005 | Blood | 1131 | 19F | ERS553501 | Female | 1 |
| C15951 | SC9 | 2007 | CSF | 1131 | 19F | ERS399094 | Female | 13 |
| D48309 | SC9 | 2008 | Blood | 1131 | 19F | ERS399095 | Male | 4 |
| B19661 | SC9 | 2009 | CSF | 361 | 19F | ERS050426 | Male | 60 |
| D36663 | SC9 | 2006 | Blood | 361 | 23F | ERS215013 | Male | 1 |
| D27133 | SC9 | 2004 | Blood | 361 | 35B | ERS553415 |  |  |
| D38253 | SC9 | 2006 | Blood | 361 | 35B | ERS625388 | Female | 1 |
| D47335 | SC9 | 2008 | Blood | 361 | 35B | ERS006754 | Female |  |
| D38165 | SC10 | 2006 | Blood | 9544 | 46 | ERS214965 | Female | 4 |
| C17274 | SC10 | 2007 | CSF | 9544 | 46 | ERS553388 | Female |  |
| C23437 | SC10 | 2010 | CSF | 9544LV | 46 | ERS553489 |  | 12 |
| C18004 | SC10 | 2008 | CSF | 9544LV | 12A | ERS006740 | Male | 11 |
| C9536 | SC10 | 2004 | CSF | 989 | 12B | ERS006725 | Male |  |
| A29943 | SC10 | 2004 | Blood | 989 | 12B | ERS012097 | Female | 30 |
| A31131 | SC10 | 2004 | Blood | 989 | 12F | ERS012099 | Male | 60 |
| B9437 | SC10 | 2004 | CSF | 989LV | 12F | ERS012107 | Male | 36 |
| A31198a | SC10 | 2004 | Blood | 989 | 12F | ERS050338 | Female | 29 |
| B13048 | SC10 | 2005 | CSF | 989LV | 12F | ERS003548 | Male | 42 |
| A36782 | SC10 | 2005 | Blood | 989LV | 12F | ERS050359 | Female | 26 |
| A37837 | SC10 | 2005 | Blood | 989 | 12F | ERS050368 | Female | 28 |
| A43416 | SC10 | 2006 | Blood | 989LV | 12F | ERS050377 |  |  |
| C15284 | SC10 | 2006 | CSF | 989 | 12F | ERS026056 |  | 10 |
| B15249 | SC10 | 2006 | CSF | 989 | 12F | ERS012076 | Female | 20 |
| D38612 | SC10 | 2006 | Blood | 6839LV | 12F | ERS006755 | Female |  |
| B17731 | SC10 | 2008 | CSF | 989 | 12F | ERS006802 | Female | 18 |
| A52232 | SC10 | 2008 | Blood | 989LV | 12F | ERS050392 | Male | 23 |
| B18494 | SC10 | 2008 | CSF | 989LV | 12F | ERS050422 | Male | 23 |
| C18919 | SC10 | 2008 | CSF | 989 | 12F | ERS050466 | Female | 3 |
| C19054 | SC10 | 2008 | CSF | 989 | 12F | ERS050469 | Male | 7 |
| A56826 | SC10 | 2009 | Blood | 989 | 12F | ERS050403 | Male | 34 |
| 1005348 | SC10 | 2010 | CSF | 989 | 12F | ERS625652 | Male | 35 |
| 1002751 | SC10 | 2010 | Blood | 3377 | 12F | ERS625658 | Male | 32 |
| A59302 | SC10 | 2010 | Blood | 989 | 12F | ERS215004 | Female | 26 |
| C23056 | SC10 | 2010 | CSF | 989 | 9A | ERS625698 | Male | 3 |
| C10592 | SC11 | 2004 | CSF | 8672 | 7A/F | ERS024519 | Male |  |
| A29030 | SC11 | 2004 | Blood | 8672 | 7A/F | ERS050332 | Female | 35 |
| A32849 | SC11 | 2004 | Blood | 8672 | 7A/F | ERS050347 | Male | 33 |
| D23780 | SC11 | 2004 | Blood | 8672 | 7A/F | ERS012111 |  |  |
| D31870 | SC11 | 2005 | Blood | 8672 | 7A/F | ERS006729 |  | 8 |
| A36949 | SC11 | 2005 | Blood | 8672 | 7A/F | ERS012130 | Female | 60 |
| D34296 | SC11 | 2005 | Blood | 8672 | 7A/F | ERS012144 | Male |  |
| B14208 | SC11 | 2006 | CSF | 8672 | 7A/F | ERS008344 | Male | 59 |
| A41543 | SC11 | 2006 | Blood | 8672 | 7A/F | ERS012065 |  |  |
| D38756 | SC11 | 2006 | Blood | 10675 | 7A/F | ERS553656 | Male | 2 |
| A42552 | SC11 | 2006 | Blood | 10675 | 7A/F | ERS050374 |  |  |
| C17622 | SC11 | 2008 | CSF | 8672 | 7A/F | ERS625416 |  |  |
| D47367 | SC11 | 2008 | Blood | 8672 | 7A/F | ERS006753 | Male |  |
| A49600 | SC11 | 2008 | Blood | 8672 | 7A/F | ERS006796 | Female | 35 |
| A51189 | SC11 | 2008 | Blood | 8672 | 7A/F | ERS050387 | Male | 40 |
| B18408 | SC11 | 2008 | CSF | 8672 | 7A/F | ERS050421 | Male | 21 |
| C18075 | SC11 | 2008 | CSF | 8672 | 7A/F | ERS050460 | Female |  |
| A52018 | SC11 | 2008 | Blood | 8672 | 7A/F | ERS008334 | Male | 22 |
| B18374 | SC11 | 2008 | CSF | 8672 | 7A/F | ERS008348 | Male | 22 |
| A55213 | SC11 | 2009 | Blood | 8672 | 7A/F | ERS398987 | Male | 40 |
| B19805 | SC11 | 2009 | CSF | 8672LV | 7A/F | ERS050428 | Female | 54 |
| C20505 | SC11 | 2009 | CSF | 8672 | 7A/F | ERS050471 | Male | 4 |
| A57916 | SC11 | 2010 | Blood | 8672 | 7A/F | ERS625713 | Female | 28 |
| A58657 | SC11 | 2010 | Blood | 9531 | 9A | ERS215000 | Female | 58 |
| A42335 | SC12 | 2006 | Blood | 5435 | 3 | ERS006794 |  |  |
| C9873 | SC12 | 2004 | CSF | 63 | 14 | ERS553405 | Female |  |
| D25696 | SC12 | 2004 | Blood | 63 | 14 | ERS006736 | Female |  |
| C9938 | SC12 | 2004 | CSF | 63 | 14 | ERS008358 | Male |  |
| A28640 | SC12 | 2004 | Blood | 63 | 14 | ERS012092 | Female | 27 |
| D26870 | SC12 | 2004 | Blood | 63 | 14 | ERS214918 |  |  |
| B12589 | SC12 | 2005 | CSF | 63 | 14 | ERS012136 | Male | 37 |
| A37501 | SC12 | 2005 | Blood | 63 | 14 | ERS050367 | Female | 32 |
| D39268 | SC12 | 2006 | Blood | 63 | 14 | ERS625406 | Male | 5 |
| D38588 | SC12 | 2006 | Blood | 2678 | 14 | ERS006724 | Female | 2 |
| A41722 | SC12 | 2006 | Blood | 63 | 14 | ERS012066 |  |  |
| C17361 | SC12 | 2007 | CSF | 63 | 14 | ERS024522 | Male |  |
| C18913 | SC12 | 2008 | CSF | 63 | 14 | ERS050465 | Female |  |
| C18952 | SC12 | 2008 | CSF | 63 | 14 | ERS050467 | Female | 7 |
| C21008 | SC12 | 2009 | CSF | 63 | 14 | ERS050477 | Female | 9 |
| D51814 | SC12 | 2009 | Blood | 63 | 14 | ERS214938 | Female | 3 |
| B22581 | SC12 | 2010 | CSF | 63 | 14 | ERS214989 | Male | 38 |
| A50302 | SC12 | 2008 | Blood | 9171LV | 37 | ERS006798 | Female | 38 |
| A30615 | SC12 | 2004 | Blood | 10608 | 33A | ERS008319 | Female | 24 |
| B9632 | SC12 | 2004 | CSF | 10608 | 33A | ERS008350 | Male | 24 |
| B15143 | SC13 | 2006 | CSF | 10868LV | 8 | ERS012074 | Female | 31 |
| D47510 | SC13 | 2008 | Blood | 10868LV | 8 | ERS024532 | Male | 13 |
| A52597 | SC13 | 2008 | Blood | 10868 | 8 | ERS003582 | Female | 38 |
| D28303 | SC13 | 2004 | Blood | 9526 | 17F | ERS553387_ | Female |  |
| D28303 | SC13 | 2004 | Blood | 9526 | 17F | ERS553387 | Female |  |
| B11670 | SC13 | 2005 | CSF | 9926 | 17F | ERS050412 | Male |  |
| A59717 | SC13 | 2010 | Blood | 9926 | 17F | ERS399000 | Female | 49 |
| A59564 | SC13 | 2010 | Blood | 9526 | 17F | ERS214995 | Female | 29 |
| D28531 | SC13 | 2004 | Blood | 9537LV | 18A | ERS006818 |  | 1 |
| D46797 | SC13 | 2008 | Blood | 9537LV | 18A | ERS008364 | Male | 6 |
| C21085 | SC13 | 2009 | CSF | 9537LV | 18A | ERS050479 | Male | 8 |
| A59818 | SC13 | 2010 | Blood | 9537 | 18A | ERS625602 | Male |  |
| A59815 | SC13 | 2010 | Blood | 9537 | 18A | ERS214986 | Male | 26 |
| A29858 | SC13 | 2004 | Blood | 9523 | 18B/C | ERS006790 | Female | 33 |
| D33733 | SC13 | 2005 | Blood | 9523 | 18B/C | ERS008361 | Male |  |
| C12675a | SC13 | 2005 | CSF | 9523 | 18B/C | ERS050452 | Male |  |
| A40807 | SC13 | 2006 | Blood | 9523 | 18B/C | ERS012062 |  |  |
| C17490 | SC13 | 2007 | CSF | 9523 | 18B/C | ERS214943 | Male | 3 |
| B19554 | SC13 | 2009 | CSF | 9523 | 18B/C | ERS050425 | Male | 25 |
| 1006938 | SC13 | 2010 | CSF | 9523 | 18B/C | ERS625717 | Female |  |
| C23125 | SC13 | 2010 | CSF | 9523 | 18B/C | ERS050482 | Female | 10 |
| C20960 | SC13 | 2009 | CSF | 9536 | 18F | ERS214959 | Female | 9 |
| C21045 | SC13 | 2009 | CSF | 9536 | 25A/F | ERS399015 | Female | 10 |
| A31265 | SC13 | 2004 | Blood | 5396 | 35B | ERS050339 | Male | 37 |
| B14721 | SC13 | 2006 | CSF | 5396 | 35B | ERS012071 | Male | 31 |
| B19728 | SC13 | 2009 | CSF | 5396 | 35B | ERS398976 | Male | 68 |
| B19728 | SC13 | 2009 | CSF | 5396 | 35B | ERS050427 | Female | 68 |
| A51119 | SC13 | 2008 | Blood | 9926 | 6A | ERS050386 |  | 47 |
| C17711 | SC13 | 2008 | CSF | 7053 | 7C | ERS050458 | Female |  |
| C10300 | SC13 | 2004 | CSF | 1871 | 9A | ERS024518 | Male | 6 |
| B9521 | SC13 | 2004 | CSF | 1871 | 9A | ERS012108 | Male | 24 |
| A34562 | SC13 | 2005 | Blood | 1871 | 9A | ERS012124 | Male | 47 |
| B18747 | SC13 | 2008 | CSF | 1871 | 9A | ERS003579 | Male | 44 |
| B9734 | SC14 | 2004 | CSF | 10633LV | 11D | ERS012110 | Female | 24 |
| A51546a | SC14 | 2008 | Blood | 10568LV | 11D | ERS003587 | Male | 30 |
| D27950 | SC14 | 2004 | Blood | 10761LV | 15A | ERS050495 | Female | 1 |
| D29418 | SC14 | 2005 | Blood | 10621 | 19A | ERS553373 |  | 1 |
| D32289 | SC14 | 2005 | Blood | 5393LV | 19F | ERS026040 |  |  |
| D29418 | SC14 | 2005 | Blood | 10621 | 19A | ERS553373 |  | 1 |
| B12658 | SC14 | 2005 | CSF | 10568LV | 19F | ERS012137 | Male | 26 |
| A41848 | SC14 | 2006 | Blood | 10568LV | 19F | ERS012067 |  |  |
| A29167 | SC14 | 2004 | Blood | 5902 | 9A | ERS012096 | Male | 30 |
| A30277 | SC14 | 2004 | Blood | 5902 | 9A | ERS012098 | Female | 21 |
| B10027 | SC14 | 2004 | CSF | 5902 | 9A | ERS012100 | Male | 41 |
| D30974 | SC14 | 2005 | Blood | 10874 | 9A | ERS553391 |  | 4 |
| D30974 | SC14 | 2005 | Blood | 10874 | 9A | ERS024513 |  | 4 |
| D34505 | SC14 | 2005 | Blood | 5902 | 9A | ERS214946 | Male | 3 |
| C17094 | SC14 | 2007 | CSF | 5902 | 9A | ERS399031 | Female | 12 |
| A59679 | SC14 | 2010 | Blood | 5399LV | 9A | ERS399021 | Female | 37 |
| D29043 | SC15 | 2004 | Blood | 9532 | 6A | ERS399311 | Female |  |
| D27140 | SC15 | 2004 | Blood | 10671 | 6A | ERS553579 | Female |  |
| A31122 | SC15 | 2004 | Blood | 9533 | 6A | ERS050336 | Male |  |
| A34964 | SC15 | 2005 | Blood | 9532 | 6A | ERS006775 | Male | 14 |
| D33275 | SC15 | 2005 | Blood | 9533 | 6A | ERS012143 | Male | 1 |
| D36051 | SC15 | 2005 | Blood | 9532 | 6A | ERS012145 | Female | 3 |
| C12480a | SC15 | 2005 | CSF | 9533 | 6A | ERS050451 | Male | 1 |
| C13356 | SC15 | 2005 | CSF | 9532 | 6A | ERS214969 | Male | 3 |
| D36355 | SC15 | 2006 | Blood | 2285 | 6A | ERS026049 | Male |  |
| C15155 | SC15 | 2006 | CSF | 2285 | 6A | ERS214977 | Male | 4 |
| D43108 | SC15 | 2007 | Blood | 9915 | 6A | ERS553392 |  | 4 |
| C17457 | SC15 | 2007 | CSF | 2285 | 6A | ERS553499 | Female | 1 |
| C17415 | SC15 | 2007 | CSF | 9532 | 6A | ERS625396 | Male | 2 |
| C16950 | SC15 | 2007 | CSF | 9532 | 6A | ERS625414 | Female | 1 |
| C18673 | SC15 | 2008 | CSF | 2285 | 6A | ERS006739 | Female | 1 |
| D46184 | SC15 | 2008 | Blood | 9575LV | 6A | ERS006757 | Female | 1 |
| A55352 | SC15 | 2009 | Blood | 9532 | 6A | ERS398965 | Male | 22 |
| D53427 | SC15 | 2009 | Blood | 9506 | 6A | ERS214937 | Female | 1 |
| B22036 | SC15 | 2010 | CSF | 9915 | 6A | ERS399078 | Male | 38 |
| D27810 | SC16 | 2004 | Blood | 10583 | 12B | ERS399335 | Male |  |
| B11223 | SC16 | 2005 | CSF | 10583 | 12B | ERS012132 | Female | 22 |
| C15275 | SC16 | 2006 | CSF | 10583 | 12B | ERS625413 | Male | 1 |
| A41112 | SC16 | 2006 | Blood | 10583 | 12B | ERS050371 |  |  |
| C14971 | SC16 | 2006 | CSF | 10583 | 12B | ERS050457 | Female | 9 |
| D38557 | SC16 | 2006 | Blood | 10583 | 12B | ERS050497 | Male |  |
| D49723 | SC16 | 2008 | Blood | 10583 | 12B | ERS625407 |  | 1 |
| B18037 | SC16 | 2008 | CSF | 10583 | 12B | ERS006801 | Male | 22 |
| C12871 | SC17 | 2005 | CSF | 2053 | 13 | ERS398853 | Female | 1 |
| D38559 | SC17 | 2006 | Blood | 2053 | 13 | ERS398911 | Male | 2 |
| C14650 | SC17 | 2006 | CSF | 2053 | 13 | ERS625389 |  | 1 |
| D38834 | SC17 | 2006 | Blood | 2053 | 13 | ERS012080 | Female | 2 |
| A39048 | SC17 | 2006 | Blood | 2053 | 13 | ERS012146 |  |  |
| D39094 | SC17 | 2006 | Blood | 2053 | 13 | ERS050637 | Male |  |
| C14277 | SC17 | 2006 | CSF | 2053 | 13 | ERS050456 | Female | 2 |
| C16492 | SC17 | 2007 | CSF | 2053 | 13 | ERS399346 | Male | 1 |
| B18487 | SC17 | 2008 | CSF | 2053 | 13 | ERS003585 | Female | 30 |
| C22343 | SC17 | 2010 | CSF | 2053 | 13 | ERS398835 | Male | 1 |
| B21503 | SC17 | 2010 | CSF | 2053 | 13 | ERS050436 | Male | 74 |
| A36611 | SC17 | 2005 | Blood | 5847LV | 31 | ERS050357 | Female | 34 |
| A51396 | SC17 | 2008 | Blood | 5847LV | 31 | ERS003568 | Male | 40 |
| A51397 | SC17 | 2008 | Blood | 5847LV | 31 | ERS003581 | Female | 33 |
| C11624 | SC17 | 2005 | CSF | 9570 | 19B | ERS398943 |  | 2 |
| C10123 | SC18 | 2004 | CSF | 9936 | 15B/C | ERS398986 |  |  |
| B10622 | SC18 | 2004 | CSF | 2773LV | 15B/C | ERS012102 | Female | 29 |
| C13233 | SC18 | 2005 | CSF | 9572 | 15B/C | ERS625398 | Male | 3 |
| C14376 | SC18 | 2006 | CSF | 9572LV | 15B/C | ERS006732 | Male | 7 |
| C14132 | SC18 | 2006 | CSF | 9936 | 15B/C | ERS006735 | Female | 2 |
| C19863 | SC18 | 2008 | CSF | 9936 | 15B/C | ERS398900 | Female | 2 |
| C18113 | SC18 | 2008 | CSF | 9572 | 15B/C | ERS050461 | Female | 8 |
| C20393 | SC18 | 2009 | CSF | 2773LV | 15B/C | ERS050470 | Male | 7 |
| B21982 | SC18 | 2010 | CSF | 9936 | 15B/C | ERS050442 | Male | 26 |
| A59464 | SC18 | 2010 | Blood | 9936 | 18B/C | ERS625690 | Male |  |
| A55557 | SC18 | 2009 | Blood | 9936 | 22A/F | ERS553507 |  |  |
| D33615 | SC18 | 2005 | Blood | 9572 | 6A | ERS398838 | Female |  |
| D25736 | SC19 | 2004 | Blood | 2715 | 19F | ERS214988 | Female |  |
| D35045 | SC19 | 2005 | Blood | 347 | 19F | ERS215005 | Female | 1 |
| D38708 | SC19 | 2006 | Blood | 347 | 19F | ERS050636 | Male |  |
| D38708 | SC19 | 2006 | Blood | 347 | 19F | ERS214999 | Female | 1 |
| C14508 | SC19 | 2006 | CSF | 347 | 19F | ERS215015 | Male | 1 |
| C16847 | SC19 | 2007 | CSF | 347 | 19F | ERS006742 | Male |  |
| D47322 | SC19 | 2008 | Blood | 347 | 19F | ERS006756 | Female | 12 |
| B20894 | SC19 | 2009 | CSF | 347 | 19F | ERS399339 | Male | 21 |
| A54575 | SC19 | 2009 | Blood | 347 | 19F | ERS050401 | Female | 45 |
| C23342 | SC19 | 2010 | CSF | 347 | 19F | ERS399349 | Female | 6 |
| D57286 | SC19 | 2010 | Blood | 9930 | 19F | ERS214976 | Male | 6 |
| D26844 | SC20 | 2004 | Blood | 2213 | 4 | ERS553422 | Female |  |
| A32279 | SC20 | 2004 | Blood | 2213 | 4 | ERS050344 | Female | 44 |
| D31058 | SC20 | 2005 | Blood | 2213 | 4 | ERS399276 | Female |  |
| C11571 | SC20 | 2005 | CSF | 2213 | 4 | ERS553577 |  | 1 |
| A33922 | SC20 | 2005 | Blood | 2213 | 4 | ERS012119 | Female | 29 |
| A36646 | SC20 | 2005 | Blood | 2213 | 4 | ERS050358 | Female | 58 |
| C13751 | SC20 | 2006 | CSF | 2213 | 4 | ERS398800 | Female | 1 |
| C14560 | SC20 | 2006 | CSF | 2213 | 4 | ERS006737 | Female | 8 |
| A43965 | SC20 | 2006 | Blood | 2213 | 4 | ERS008332 |  |  |
| A47266 | SC20 | 2007 | Blood | 2213 | 4 | ERS003572 | Male |  |
| A50227 | SC20 | 2008 | Blood | 2213 | 4 | ERS050385 | Male | 40 |
| A57013 | SC20 | 2009 | Blood | 2213 | 4 | ERS625662 | Female | 31 |
| A56917 | SC20 | 2009 | Blood | 2213 | 4 | ERS625704 | Female | 51 |
| D51832 | SC20 | 2009 | Blood | 2213 | 4 | ERS214979 | Male | 9 |
| A59776 | SC20 | 2010 | Blood | 2213 | 4 | ERS214991 | Male | 33 |
| B22515 | SC20 | 2010 | CSF | 9554 | 4 | ERS214993 | Female | 23 |
| C9864 | SC21 | 2004 | CSF | 2987 | 6A | ERS625418 | Female |  |
| D24847 | SC21 | 2004 | Blood | 2987 | 6A | ERS012112 | Female |  |
| C9909 | SC21 | 2004 | CSF | 2987 | 6A | ERS215007 |  |  |
| C10321 | SC21 | 2004 | CSF | 2987 | 6A | ERS553394 | Female |  |
| C13117 | SC21 | 2005 | CSF | 2987 | 6A | ERS398935 | Female | 2 |
| D34462 | SC21 | 2005 | Blood | 10596 | 6A | ERS553569 | Male | 1 |
| D30541 | SC21 | 2005 | Blood | 2987 | 6A | ERS553386 |  | 2 |
| D30541 | SC21 | 2005 | Blood | 2987 | 6A | ERS553386 |  | 2 |
| A37893 | SC21 | 2005 | Blood | 2987 | 6A | ERS008328 | Male |  |
| B12772 | SC21 | 2005 | CSF | 2987 | 6A | ERS008343 | Female | 40 |
| C12495 | SC21 | 2005 | CSF | 2987 | 6A | ERS008352 | Female | 8 |
| D33293 | SC21 | 2005 | Blood | 2987 | 6A | ERS008360 |  | 8 |
| A36891 | SC21 | 2005 | Blood | 10596 | 6A | ERS050360 | Female | 32 |
| C11950 | SC21 | 2005 | CSF | 2987 | 6A | ERS008351 |  |  |
| D37499 | SC21 | 2006 | Blood | 2987 | 6A | ERS553376 | Female | 3 |
| D37725 | SC21 | 2006 | Blood | 2987LV | 6A | ERS006726 | Male | 5 |
| A42000 | SC21 | 2006 | Blood | 2987 | 6A | ERS012068 |  |  |
| C14215 | SC21 | 2006 | CSF | 2987 | 6A | ERS012078 | Male | 11 |
| D46329 | SC21 | 2008 | Blood | 2987LV | 6A | ERS012090 | Female | 1 |
| D51924 | SC21 | 2009 | Blood | 9496 | 6A | ERS214934 | Female | 2 |
| C10270 | SC22 | 2004 | CSF | 9476 | 2 | ERS553407 |  |  |
| C22387 | SC22 | 2010 | CSF | 9476 | 2 | ERS214990 | Male | 13 |
| A37855 | SC22 | 2005 | Blood | 1004LV | 4 | ERS006786 | Male |  |
| B20065 | SC22 | 2009 | CSF | 2234 | 8 | ERS050430 | Male | 24 |
| D32344 | SC22 | 2005 | Blood | 10595 | 13 | ERS026043 |  | 7 |
| A33054 | SC22 | 2005 | Blood | 3548 | 31 | ERS012114 | Male |  |
| D24854 | SC22 | 2004 | Blood | 9524 | 34 | ERS399013 | Female |  |
| D31827 | SC22 | 2005 | Blood | 9546LV | 34 | ERS008359 |  |  |
| B18762 | SC22 | 2008 | CSF | 9546LV | 34 | ERS008349 | Male | 36 |
| B11188 | SC22 | 2005 | CSF | 9445 | 45 | ERS003554 | Female | 40 |
| B11811 | SC22 | 2005 | CSF | 9445 | 45 | ERS003556 | Male | 35 |
| A43382 | SC22 | 2006 | Blood | 9445 | 45 | ERS006805 |  |  |
| B15467 | SC22 | 2007 | CSF | 9445 | 45 | ERS003574 | Male | 24 |
| B18210a | SC22 | 2008 | CSF | 9445 | 45 | ERS050419 | Female | 30 |
| C20777 | SC22 | 2009 | CSF | 9553 | 15B/C | ERS398822 | Female | 1 |
| A51905 | SC22 | 2008 | Blood | 6030 | 16F | ERS050389 | Female | 42 |
| A60380 | SC22 | 2010 | Blood | 6030 | 16F | ERS625931 | Male | 46 |
| B21977 | SC22 | 2010 | CSF | 5326LV | 16F | ERS625693 | Male | 46 |
| B21950 | SC22 | 2010 | CSF | 6030 | 16F | ERS050440 | Female | 17 |
| C10281 | SC22 | 2004 | CSF | 102 | 18B/C | ERS024520 | Male | 4 |
| D28166 | SC22 | 2004 | Blood | 102 | 18B/C | ERS012113 | Female |  |
| C13260 | SC22 | 2005 | CSF | 5266LV | 18B/C | ERS026048 | Male |  |
| C16094 | SC22 | 2007 | CSF | 9522 | 18B/C | ERS215017 | Male | 1 |
| C19000 | SC22 | 2008 | CSF | 2927 | 18B/C | ERS050468 | Male | 6 |
| B19190 | SC22 | 2009 | CSF | 9484LV | 18B/C | ERS050424 | Female | 27 |
| C20742 | SC22 | 2009 | CSF | 5266 | 18B/C | ERS050473 | Male | 1 |
| D49793 | SC22 | 2009 | Blood | 2927 | 18B/C | ERS214909 | Male | 1 |
| C23050 | SC22 | 2010 | CSF | 5266 | 18B/C | ERS625723 | Female | 1 |
| C23171 | SC22 | 2010 | CSF | 9903 | 18B/C | ERS050483 | Female | 1 |
| D56178 | SC22 | 2010 | Blood | 9903 | 18B/C | ERS214936 | Male | 1 |
| D56349 | SC22 | 2010 | Blood | 5266 | 18B/C | ERS214983 | Male | 13 |
| D40313 | SC22 | 2006 | Blood | 5262 | 19A | ERS398804 | Male | 1 |
| C16554 | SC22 | 2007 | CSF | 847 | 19A | ERS399275 | Female | 1 |
| C16949 | SC22 | 2007 | CSF | 5080 | 19A | ERS214947 | Male | 4 |
| D50597 | SC22 | 2009 | Blood | 847 | 19A | ERS214945 | Male | 1 |
| A28899 | SC22 | 2004 | Blood | 5266 | 22A/F | ERS006791 | Male | 38 |
| A55235 | SC22 | 2009 | Blood | 9546 | 22A/F | ERS553375 | Female | 35 |
| A53861 | SC22 | 2009 | Blood | 9546 | 22A/F | ERS050398 | Male | 32 |
| B20084 | SC22 | 2009 | CSF | 9546 | 22A/F | ERS050431 | Female | 32 |
| C9268 | SC22 | 2004 | CSF | 5080 | 23A | ERS553395 |  |  |
| D24904 | SC22 | 2004 | Blood | 5080 | 23A | ERS024515 | Male |  |
| D26572 | SC22 | 2004 | Blood | 10609 | 23F | ERS553397 | Female |  |
| C10016 | SC22 | 2004 | CSF | 10609 | 23F | ERS553380 | Male |  |
| D33678 | SC22 | 2005 | Blood | 9491 | 23F | ERS553409 | Male | 2 |
| A58946 | SC22 | 2010 | Blood | 9491 | 23F | ERS399285 | Male | 35 |
| 1005792 | SC22 | 2010 | Blood | 9491 | 23F | ERS214948 |  | 1 |
| B19944 | SC22 | 2009 | CSF | 5079 | 24A | ERS050429 | Female | 47 |
| A57882 | SC22 | 2010 | Blood | 5079 | 24A | ERS625709 | Female | 36 |
| A29265 | SC22 | 2004 | Blood | 5077LV | 24F | ERS050333 | Male | 37 |
| A32497 | SC22 | 2004 | Blood | 5077 | 24F | ERS050345 | Male | 45 |
| C12879a | SC22 | 2005 | CSF | 5077 | 24F | ERS050454 | Male | 13 |
| B8923 | SC22 | 2004 | CSF | 5604 | 25A/F | ERS012104 | Male |  |
| A42501 | SC22 | 2006 | Blood | 2650 | 25A/F | ERS050373 |  |  |
| A47814 | SC22 | 2007 | Blood | 2650 | 25A/F | ERS003571 | Male | 30 |
| A49649 | SC22 | 2008 | Blood | 2650 | 25A/F | ERS050384 | Male | 51 |
| A56847 | SC22 | 2009 | Blood | 105 | 25A/F | ERS399023 | Female | 48 |
| B20631 | SC22 | 2009 | CSF | 2650 | 25A/F | ERS050434 | Female |  |
| D28503 | SC22 | 2004 | Blood | 7063 | 6A | ERS553419 |  |  |
| A36530 | SC22 | 2005 | Blood | 7063 | 6A | ERS008325 | Male | 31 |
| B12226 | SC22 | 2005 | CSF | 7063 | 6A | ERS008340 | Female | 31 |
| D37395 | SC22 | 2006 | Blood | 3207 | 6A | ERS398869 | Female | 1 |
| A47073 | SC22 | 2007 | Blood | 5412 | 6A | ERS008333 | Female | 40 |
| D47292 | SC22 | 2008 | Blood | 8267 | 6A | ERS024530 | Male |  |
| A51983 | SC22 | 2008 | Blood | 3207LV | 6A | ERS050391 | Male | 29 |
| C21075 | SC22 | 2009 | CSF | 8267 | 6A | ERS399270 | Female |  |
| D54560 | SC22 | 2010 | Blood | 914 | 6A | ERS625960 | Female |  |
| A58785 | SC22 | 2010 | Blood | 8267LV | 6A | ERS050406 | Male | 32 |
| C22246 | SC22 | 2010 | CSF | 914 | 6A | ERS214915 | Male | 2 |
| B21600 | SC22 | 2010 | CSF | 7653LV | 6D | ERS050438 | Male | 29 |
| B17790 | SC22 | 2008 | CSF | 3544 | 7A/F | ERS012089 | Female | 35 |
| A33813 | SC22 | 2005 | Blood | 5266LV | 9A | ERS012117 | Female | 20 |
| A34292 | SC22 | 2005 | Blood | 5778 | 9A | ERS012123 | Female | 29 |
| B11855 | SC22 | 2005 | CSF | 5778 | 9A | ERS012135 | Female | 27 |
| D43004 | SC22 | 2007 | Blood | 706 | 9A | ERS214951 |  | 2 |
| C20650 | SC22 | 2009 | CSF | 5778LV | 9A | ERS050472 | Male | 1 |
| A57226 | SC22 | 2009 | Blood | 5778 | 9A | ERS625694 | Male |  |
| B20776 | SC22 | 2009 | CSF | 4221LV | 9A | ERS050435 | Male |  |
| C20932 | SC22 | 2009 | CSF | 9473 | 9A | ERS050476 | Male | 4 |
| D51826 | SC22 | 2009 | Blood | 9473 | 9A | ERS214967 | Female | 14 |
| D54934 | SC22 | 2010 | Blood | 706 | 9A | ERS625730 | Male | 8 |
| A34594 | SC22 | 2005 | Blood | 9471 | 9L | ERS012126 |  | 22 |
| D32101 | SC22 | 2005 | Blood | 9471 | 9L | ERS215003 | Female |  |
| C14257 | SC22 | 2006 | CSF | 9471 | 9L | ERS050455 | Female |  |
| D41088 | SC22 | 2007 | Blood | 4914LV | 9L | ERS006750 | Male |  |
| C18159 | SC22 | 2008 | CSF | 9460 | 9L | ERS398854 | Female | 1 |
